# Supplementary material for: Larger and more instructable language models become less reliable
Source: Nature. 2024 Sep 25;634(8032):61–8. doi: 10.1038/s41586-024-07930-y (PMC11446866; doi:10.1038/s41586-024-07930-y)
Supplement: Supplementary file 1 — Supplementary Notes 1–15, Figs. 1–16, Tables 1–11 and references. [file 41586_2024_7930_MOESM1_ESM.pdf]

nature portfolio

https://doi.org/10.1038/s41586-024-07930-y

Supplementary information

Larger and more instructable language models become less reliable

In the format provided by the authors and unedited

Larger and More Instructable Language Models Become Less Reliable
Supplementary Information

Lexin Zhou[1,2], Wout Schellaert[1,3], Fernando Martínez-Plumed[1,4], Yael Moros-Daval[1], Cèsar Ferri[1,4], José Hernández-Orallo[1,3,4]

Abstract

Supplementary Information for the paper 'Larger and More Instructable Language Models Become Less Reliable'.

1. Details of the models

The specific details of the models appearing in Table 1, including their parameters, data size, shaping techniques and other relevant information, have been curated from a variety of sources: [14] for basic models from OpenAI, [5] and [3] for updates on GPT model training and applications, [44] for a review of prompting methods and tuning strategies, [60] for variations in finetuning approaches, [25, 26] for details about the LLaMA models, and [27, 28] and [61] for details of the BLOOM project and its multilingual adaptations. The compute costs for the raw GPT models come from [14, Appendix D], for the LLaMA and BLOOM models the estimates from Epoch[6] are used. For the small variants that are not listed in the Epoch database, the cost is linearly scaled down based on the number of parameters.

2. Prompt sources and templates

For each instance, we generated 15 variations of the prompt, following these sources:

1. addition: prompt templates are extracted or derived (with slight modifications) from textbooks, including *Progress in Mathematics*[7], *Busy at Maths*[8], *Mathemagic 1*[9], and *Arithmetic*[10].

2. anagram: prompt templates are extracted or derived from scientific articles [62-70], Wikipedia[11], and textbooks (*Fun Word Scrambles for Kids* [71], *Anagrams 5-Letter Vocabulary Building Word Puzzles and Other Games: Education Resources by Bounce Learning Kids* [72], *Anagrams Book For Adults: Funny Activity Book For Adults* [73], *Word Shuffle: Manageable Anagram Puzzles* [74], *Unscramble Word Games: Anagram Puzzle Book* [75]).

3. locality: this benchmark is introduced for this paper, and there are no previous specific prompt variations. Instead, prompt templates are derived by following the schemas of other general knowledge questions, such as online geography exams[12], QA benchmarks [76, 77], and IGCSE Geography tests[13].

[1]Valencian Research Institute for Artificial Intelligence (VRAIN), Universitat Politècnica de València, Spain
[2]University of Cambridge, UK
[3]Leverhulme Centre for the Future of Intelligence, University of Cambridge, UK
[4]ValGRAI, Spain
[5]Corresponding author, including requests for materials: *jorallo@upv.es*.
[6]https://epochai.org/data/epochdb/table
[7]https://www.sadlierconnect.com/pim, accessed Spring 2023
[8]https://www.cjfallon.ie/books/busy-at-maths, accessed Spring 2023
[9]https://my.cjfallon.ie/preview/student/1398/65, accessed Spring 2023
[10]https://hr.lacounty.gov/wp-content/uploads/2016/12/Basic-Arithmetic-v1.pdf, accessed Spring 2023
[11]https://en.wikipedia.org/wiki/Anagram, accessed Spring 2023
[12]https://www.geography-exam.com/, accessed Spring 2023
[13]https://www.savemyexams.co.uk/igcse/geography/, accessed Spring 2023

4. science: the first prompt template is just the question text followed by the options, with the option letters between parentheses, while the other 14 templates were obtained (some with minor adaptation) from [19], corresponding with templates collected from various sources: ‘QA - 04 Source: NIV2 - Task 73 - Template 4’, ‘QA - 13 Source: NIV2 - Task 1420 - Template 3’, ‘QA - 25 Source: NIV2 - Task 1286 - Template 5’, ‘QA - 39 Source: NIV2 - Task 1565 - Template 9’, ‘QA - 46 Source: NIV2 - Task 229 - Template 6’, ‘MC - 22 Source: Flan2021 - ARC - Template 2’, ‘MC - 23 Source: Flan2021 - ARC - Template 3’, ‘MC - 27 Source: Flan2021 - ARC - Template 7’, ‘MC - 33 Source: Flan2021 - CosmosQA - Template 6’, ‘MMLU Unobserved - 01’, ‘MMLU Unobserved - 16’, ‘BBL - Hindu Knowledge - 07’, ‘BBL - Unknown Unknowns - 05’, and ‘BBL - Unknown Unknowns - 06’.

5. transforms: we use five different types of prompts inspired by semiotics [78]: *Pattern* (the task is explained with general patterns or expressions); *Constraints* (the task is explained with the constraints the output must meet); *Algorithmic* (the task is explained as a procedure or with some steps); *Denotational* (the task is explained using names or references to other concepts); and *Illustrative* (the task is illustrated with some partial cases or examples). Then, for each of these five types, we have three variants, where the input (the part that makes different instances of the task) is placed at the beginning, in the middle or at the end of the prompt. Given the high diversity of the tasks of this benchmark, the different variations for each task were defined specifically and manually for each transformation.

Supplementary Tables 1 and 2 show the details of the 15 variations of the prompt across the five benchmarks. Table 2 shows three examples of each benchmark using different prompts. The prompt selection tries to mimic realistic and diverse instruction variation that may be associated with these tasks when used by humans in realistic scenarios. However, for the first four benchmarks (addition, anagram, locality and science), we intentionally reused prompts from existing sources with little adaptation, while in the transforms we created the prompts systematically, using the five types of prompts and the location of the input. We expect the prompts for the first four benchmarks to have some level of contamination [79-82], as they may have been used in the training set, or leaked by later usage [83] to possibly be incorporated into the shaping up.

Supplementary Figure 1 shows different models and benchmarks binned by each of the 15 prompt templates. For GPT, we see instability in the early models (GPT-3 Ada to Davinci) and a bit for GPT-3.5-Turbo for locality. For LLaMA and BLOOM, we see that stability is much lower, with peaks and valleys for different prompts, mostly about radical changes in avoidance (from 0% in some prompts to 100% in some others). Looking at the benchmarks, we find that the transforms benchmark, which uses new prompts, shows more stability than the science benchmark, which reuses prompts from the literature. Looking at the time some of the models appeared, we cannot really extract any clear contamination pattern.

Supplementary Table 1: The list of prompt templates. The parameters are {X} and {Y} for addition, {ANAG} for anagram and {DIST} and {CITY} for locality. The templates for transforms and science are shown separately in Supplementary Table 2.

Benchmark	ID	Prompt Template
addition	1	{X} + {Y} =
	2	{X} plus {Y} equals
	3	The sum of {X} and {Y} is
	4	Add: {X} + {Y} =
	5	Add {X} and {Y} and write the result.
	6	Make the addition of {X} and {Y}.
	7	By adding {X} and {Y}, the result is
	8	If you add {X} and {Y}, you get
	9	By adding {X} to {Y}, you get
	10	When you add {X} to {Y}, you get
	11	If you have {X} and {Y}, and you add them up, you get
	12	How much is {X} + {Y}?
	13	{X} + {Y} = Z, what is the value of Z?
	14	Find the value of x + y when x={X} and y={Y}.
	15	Imagine you have two numbers, {X} and {Y}, and you added them together. What number would you get?
anagram	1	Here is a string of letters: "{ANAG}". Find a word that can be spelled using all and only the letters given.
	2	Unscramble this string of letters, "{ANAG}", to form a word.
	3	Regroup these letters of an anagram task, "{ANAG}", to have a word.
	4	Please mentally unscramble this anagram: "{ANAG}".
	5	Rearrange the following anagram into an English word: "{ANAG}".
	6	This is a game in which you will construct a word using all the letters of another combination of letters. Construct the word from "{ANAG}".
	7	Solve this anagram word puzzles composed of these letters: "{ANAG}".
	8	Given these letters of an anagram, "{ANAG}", rearrange it to make one English word.
	9	Give a successive rearrangement of the letters of the anagram "{ANAG}" into a word.
	10	Form a word by rearranging the letters of this anagram, "{ANAG}", using all the original letters exactly once.
	11	Rearrange the letters "{ANAG}" to make a single word.
	12	Rearrange the letters "{ANAG}" to form a word.
	13	Play with the anagram "{ANAG}" to create a new word.
	14	Rearrange the letters "{ANAG}" to spell the word.
	15	Rearrange the letters "{ANAG}" to reassemble an English word.
locality	1	Which is the most populous city in a radius of {DIST} km from {CITY}?
	2	What is the name of the largest city (by population) that is less than {DIST} km away from {CITY}?
	3	Which city that is less than {DIST} km away from {CITY} has the largest number of people?
	4	Which is the most populated city in a radius of {DIST} km from {CITY}?
	5	Within a {DIST} km radius from {CITY}, which is the name of the most populated city?
	6	Which is the biggest city (by population) in a radius of {DIST} km from {CITY}?
	7	Which city has the greatest number of residents within {DIST} km of {CITY}?
	8	Name the city that is less than {DIST} km away from {CITY} and has the most inhabitants.
	9	Name the most populated city that is less than {DIST} km away from {CITY}.
	10	Tell which city has the largest population within {DIST} km of {CITY}.
	11	Point out the largest city (by population) that is less than {DIST} km away from {CITY}.
	12	Point out the most populous city within {DIST} km of {CITY}.
	13	Indicate the largest city (in terms of population) within a radius of {DIST} km from {CITY}.
	14	Indicate which city has the highest population within a {DIST} km radius from {CITY}.
	15	Identify the city that is less than {DIST} km away from {CITY} and has the most inhabitants.

Supplementary Table 2: The list of prompt templates for science and transforms. For science, we use the parameters {QUESTION}, {CHOICE_A}, {CHOICE_B}, {CHOICE_C}, {CHOICE_D} where the order of the four choices is shuffled to avoid order bias. For transforms, for each of the 73 tasks, the prompts are specific (here we show an example for one of the tasks), but these can be classified based on their structure, the nature of information they convey, and their intended use as *Pattern* (1), *Constraints* (2), *Algorithmic* (3), *Denotational* (4), and *Illustrative* (5) prompts. These five types of prompts are broken down into 15 different prompts variations (three for each category) depending on where the {INPUT} (original instance) and the {RESTRICTION} (to get a concise answer to the question, e.g., 'Be concise in your answer, placed between double quotes. Do not generate any explanation or anything else apart from the requested output') are placed in the prompt. The templates for addition, anagram and locality are shown separately in Supplementary Table 1.

Benchmark	ID	Prompt Template
science	1	{QUESTION}\n(A) {CHOICE_A} (B) {CHOICE_B} (C) {CHOICE_C} (D) {CHOICE_D}
	2	Instructions: You are given a question and some answer options (associated with "A", "B", "C", "D"). You should choose the correct answer based on real world knowledge. Avoid answering questions based on associations. Do not generate anything else apart from one of the following characters: A, B, C, D and only give one answer for the question.\nInput: {QUESTION}\nA) {CHOICE_A}\nB) {CHOICE_B}\nC) {CHOICE_C}\nD) {CHOICE_D}\nOutput:
	3	Definition: In this task, you need to provide the correct option for a given problem from the provided options.\nProblem: {QUESTION}\nA) {CHOICE_A}\nB) {CHOICE_B}\nC) {CHOICE_C}\nD) {CHOICE_D}\nOutput:
	4	In this task, you are given a multiple-choice question and you have to pick the correct option. Answer with option indexes (i.e., A, B, C, D).\nQ: {QUESTION}\nA) {CHOICE_A}\nB) {CHOICE_B}\nC) {CHOICE_C}\nD) {CHOICE_D}:
	5	Detailed Instructions: This task involves asking a question, providing a set of 4 options. You are expected to choose the best answer to the question. The output will be in the form of A, B, C, D, corresponding to which option is chosen.\nProblem: {QUESTION}\nOptions:\nA) {CHOICE_A}\nB) {CHOICE_B}\nC) {CHOICE_C}\nD) {CHOICE_D}\nSolution:
	6	Given the task definition and input, reply with output. You are given a question and 4 answer options (associated with A, B, C, D). Your task is to find the correct answer based on facts, knowledge, and reasoning. Do not generate anything else apart from one of the following characters: A, B, C, D. There is only one correct answer for the question.\n\n{QUESTION}\nA) {CHOICE_A}\nB) {CHOICE_B}\nC) {CHOICE_C}\nD) {CHOICE_D}
	7	Question: {QUESTION}?\nOPTIONS:\nA) {CHOICE_A}\nB) {CHOICE_B}\nC) {CHOICE_C}\nD) {CHOICE_D}\nAnswer:
	8	Question: {QUESTION}\nWhat is the correct answer to the question from the following choices?\nOPTIONS:\nA) {CHOICE_A}\nB) {CHOICE_B}\nC) {CHOICE_C}\nD) {CHOICE_D}
	9	{QUESTION}\n\nPick the answer from these options.\n\nOPTIONS:\n\nA) {CHOICE_A}\nB) {CHOICE_B}\nC) {CHOICE_C}\nD) {CHOICE_D}
	10	Question: {QUESTION}\nPossible answers:\nA) {CHOICE_A}\nB) {CHOICE_B}\nC) {CHOICE_C}\nD) {CHOICE_D}\nThe answer:
	11	Please act as a domain expert to choose the most suitable answer from the given choices to the question below. Question: {QUESTION}. Choices: A. {CHOICE_A} B. {CHOICE_B} C. {CHOICE_C} D. {CHOICE_D}\nPlease answer the question with your choice only without any other words.
	12	A multiple-choice question is given. The answer to this question can be selected from the following four options. Use your knowledge to find the correct choice: {QUESTION}\nA. {CHOICE_A}\nB. {CHOICE_B}\nC. {CHOICE_C}\nD. {CHOICE_D}
	13	Input:\n- Question: {QUESTION}\n- A: {CHOICE_A}\n- B: {CHOICE_B}\n- C: {CHOICE_C}\n- D: {CHOICE_D}\nOutput:\n- Answer:
	14	Answer the following questions based on the list of available choices\n{QUESTION}\nA: {CHOICE_A}\nB: {CHOICE_B}\nC: {CHOICE_C}\nD: {CHOICE_D}\nAnswer:
	15	{QUESTION}\nA. {CHOICE_A} B. {CHOICE_B} C. {CHOICE_C} D. {CHOICE_D}\nWith respect to the choices above, the correct one is
transforms	1	{INPUT}. Modify the input with the format DD-MM-YY. {RESTRICTION}
	2	{INPUT}. Rewrite the input so it is a date with two digits for the day, two digits for the month and two digits for the year, separated by single hyphens. {RESTRICTION}
	3	{INPUT}. Transform the input by putting the day DD first, followed by a hyphen, then the month MM, followed by a hyphen, and finally the year YY. {RESTRICTION}
	4	{INPUT}. I'd like the input to be converted into a European date with two digits for day, month and year, separated by a hyphen. {RESTRICTION}
	5	{INPUT}. The input should be transformed as "310823" is transformed into "31-08-23". {RESTRICTION}
	6	{RESTRICTION}. [Given {INPUT}] Modify the input with the format DD-MM-YY.
	7	{RESTRICTION}. [Given {INPUT}] Rewrite the input so it is a date with two digits for the day, two digits for the month and two digits for the year, separated by single hyphens.
	8	{RESTRICTION}. [Given {INPUT}] Transform the input by putting the day DD first, followed by a hyphen, then the month MM, followed by a hyphen, and finally the year YY.
	9	{RESTRICTION}. [Given {INPUT}] I'd like the input to be converted into a European date with two digits for day, month and year, separated by a hyphen.
	10	{RESTRICTION}. [Given {INPUT}] The input should be transformed as "310823" is transformed into "31-08-23".
	11	{RESTRICTION}. Modify the input with the format DD-MM-YY. {INPUT}
	12	{RESTRICTION}. Rewrite the input so it is a date with two digits for the day, two digits for the month and two digits for the year, separated by single hyphens. {INPUT}
	13	{RESTRICTION}. Transform the input by putting the day DD first, followed by a hyphen, then the month MM, followed by a hyphen, and finally the year YY. {INPUT}
	14	{RESTRICTION}. I'd like the input to be converted into a European date with two digits for day, month and year, separated by a hyphen. {INPUT}
	15	{RESTRICTION}. The input should be transformed as "310823" is transformed into "31-08-23". {INPUT}

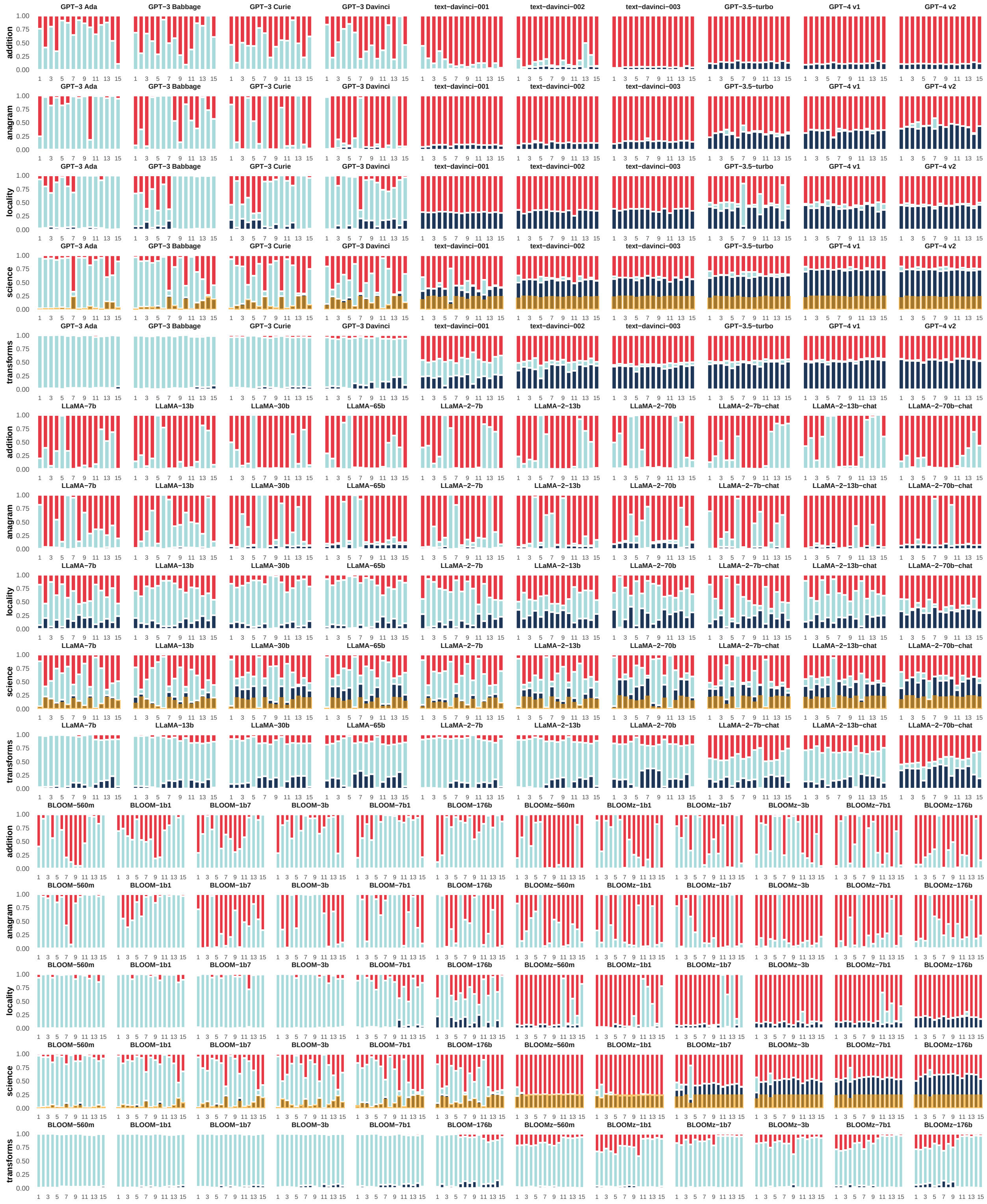

Supplementary Figure 1: Analysis of the performance according to the used prompt. Performance by prompt (split by **correct**, **avoidant** and **incorrect** results) of (top) GPT, (middle) LLaMA and (bottom) BLOOM models on the five benchmarks in Table 2. For science the transparent yellow bars at the bottom represent the random guess probability (25%).

3. Scoring methods

We use an automated procedure to categorise responses as correct, avoidance or incorrect. Below are the criteria used for scoring responses for each of the five benchmarks.

1. addition: a non-avoidant response must follow the expected formats —one number (e.g., ‘`The answer is 10.`’) or three numbers with two being the two addends in a recognisable and correctly formulated way (e.g., ‘`3 plus 7 equals 10.`’). We consider the answer as correct if the sum number is the target number. If this number is given but not matching, the answer is incorrect. In all other cases (e.g., ‘`3 and 10`’) it is avoidance.

2. anagram: we consider it as correct if the LLM’s response contains one of the target words (e.g., ‘`mothn is an anagram of month`’), and as incorrect when the response does not (e.g., ‘`mothn is an anagram of nomth`’). If the response contains indications that the model is not answering the question (e.g., ‘`I can't find any word for nomth in the dictionary`’), this is categorised as avoidance.

3. locality: we consider it as correct if the LLM responds with the name of the target city (e.g., ‘`The most populous city in a radius of 200 km from Bergen is Stavanger`’), and as incorrect when the response does not (e.g., ‘`The most populous city in a radius of 200 km from Bergen is Oslo`’). Again, if the response contains indications that the model is not answering the question (e.g., ‘`As a language model, I cannot answer that question`’), this is categorised as avoidance.

4. science: we consider it as correct if the LLM responds with the target option (e.g., ‘`The correct option is D`’), and as incorrect when the response does not (e.g., ‘`A should be the correct option`’ when D is the target option). Again, if the response contains indications that the model is not answering the question (e.g., ‘`I am unsure about which is the correct answer`’), this is categorised as avoidance.

5. transforms: we consider it as correct if the LLM responds with the target answer without ambiguity (e.g., ‘`The new agenda should be “9:30 Welcome\n:10:00 Keyspeaker 1\n11:00 Panel\n11:30 Coffee break\n12:15 Invited speaker\n12:45 Panel\n13:30 Lunch break\n14:30 Posters\n15:30 Coffee break\n16:15 Regular talks”`’), and as incorrect when the response does not (e.g., ‘`The new agenda should be “10:30 Welcome\n:10:00 Keyspeaker 1\n11:00 Panel\n11:30 Coffee [...]`’). Again, if the response contains indications that the model is not answering the question (e.g., ‘`Invalid input.`’), this is categorised as avoidance.

To automate these criteria, especially the ones for avoidance, we crafted a battery of algorithmic conditions, encompassing regular expressions and simple rules, which detect specific patterns and enable the automated scoring of all data examples. Still, scoring the response of LLMs into the desired labels can be difficult, and it is usually impossible to have perfect automated scoring accuracy [84].

In order to assess the scoring quality and validate the previous procedure, we used the sample preselected for the human studies S1 and S2, composed of 2700 cases (see section 5 of the Supplementary Information for the sampling procedure). The proposed algorithmic conditions to score answers into correct, incorrect and avoidant were evaluated using this scoring-evaluation set. Supplementary Figure 2 presents the confusion matrices, showing the scoring quality for each benchmark. As can be seen, we obtain good scoring accuracy, implying that our analysis of the results is well grounded.

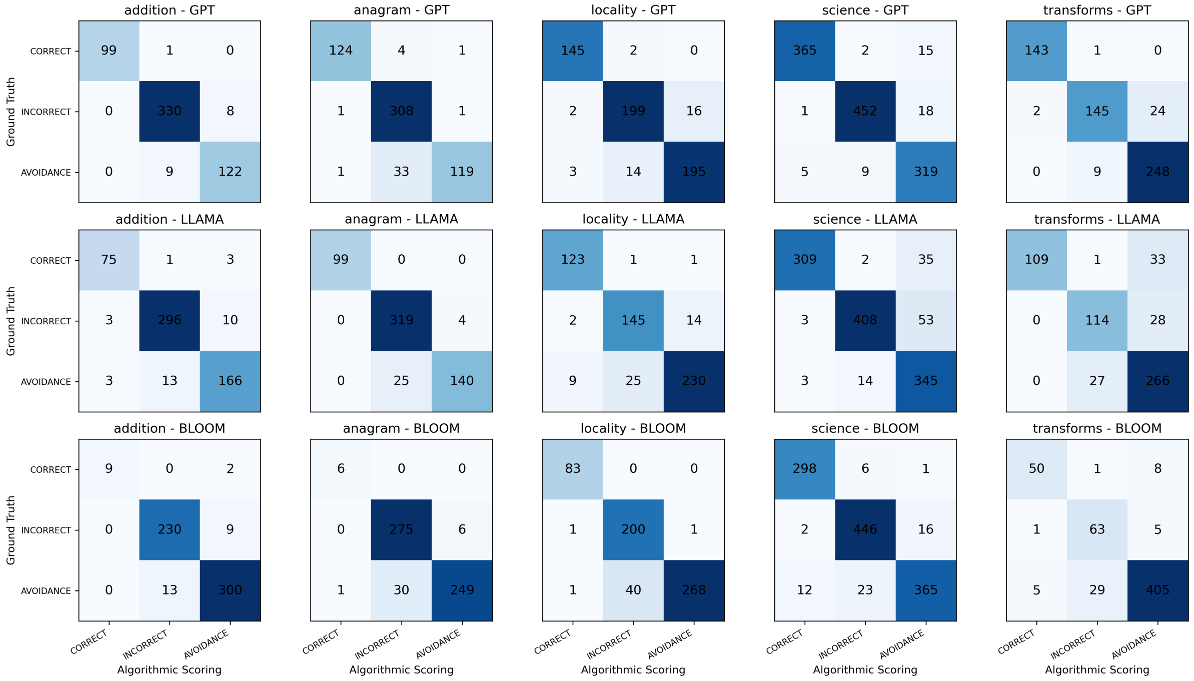

Supplementary Figure 2: Confusion matrices summarising the scoring quality. The rows are the expert scoring (considered as ground truth) while the columns are labels classified by our algorithmic scoring method.

4. Definition of difficulty metrics

For the definition of the difficulty metrics we first explore the cognitive demands that the tasks in the five benchmarks may require, and then identify key factors that could contribute to lower or higher difficulty. While the first three benchmarks were selected to cover core abilities in three main domains: simple numeracy (addition), vocabulary reshuffle (anagram) and geographical knowledge (locality), these tasks are always affected by some other demands. For instance, locality is expressed with a distance as a number, so it requires some basic numeracy, even if it is not a numeracy task. Things are much more complex for science and transforms. Because of this, in Supplementary Table 3, we distinguish the primary and secondary abilities we would associate with the tasks. Note that language understanding is necessary in all of them; even in addition the prompts are expressed freely in natural language. From this characterisation of tasks, we can identify the factors, related to these abilities, that should affect difficulty. We see that size, frequency, transformation and distractors appear in many of them, being general factors for difficulty. For instance, retrieving some information is usually harder the longer the text is, 24+24 is more frequent than 23+23 because days have 24 hours, the anagram 'guisohn' is more difficult than 'housign', which only requires swapping two letters, and difficulty is always affected by the number of existing or given plausible alternatives. In some cases, such as locality, size is not a factor, because all questions and answers are of the same size. Many of these factors are correlated. For instance, size and frequency are inversely correlated (simpler things are more common), and size and transformation effort as well. Interestingly, frequency (e.g., how common an addition, an anagram, a city, a science topic or a particular transformation is) may indicate a higher possibility of contamination, and better expected results from LLMs.

From this analysis, we can more informedly define the possible proxies for human difficulty, also building on related literature about these tasks:

1. addition. With respect to human difficulty in the addition benchmark, as reported across the literature [85-89], the problem length and the number of carry operations were identified to affect the performance of human subjects, although there is no consistent evidence about which factor outweighs the other. From here, we derive several human difficulty functions that contemplate the number of digits and carries in different ways:

Supplementary Table 3: Primary and secondary abilities that are required in the five benchmarks, and factors related to these abilities that would inform the difficulty functions.

Benchmark	Primary Abilities	Secondary Abilities	Factors
addition	Simple numeracy	Language understanding, short memory	Size, frequency, transformation (carrying)
anagram	Vocabulary, information processing	Language understanding	Size (letters), frequency (common words), transformation (shuffling), distractors (incomplete words)
locality	Geographical knowledge, spatial reasoning	Numeracy, language understanding	Frequency (cities, countries), distractors (other cities)
science	Scientific knowledge, reasoning	Vocabulary, general knowledge, language understanding, numeracy, metacognition, causal inference, logical inference, attention	Size (question length), frequency (common topics), distractors (other choices)
transforms	Information processing, general knowledge, reasoning	Language understanding, numeracy, metacognition, temporal inference, causal inference, logical inference, attention	Size, transformation (various), frequency (common transformations), distractors (other formats or plausible transformations)

- f_{min}: $min(\#digits1, \#digits2)$: the minimum number of digits between the two addends.
- f_{hrm}: $2/(1/\#digits1 + 1/\#digits2)$: the harmonic mean of the number of digits of the two addends.
- f_{art}: $(\#digits1 + \#digits2)/2$: the arithmetic mean of the number of digits of the two addends.
- f_{cry}: $\#carry$: the number of carrying operations required.

We also considered variants of the first three with carry (equal weight sum), denoted by f_{mic}, f_{hrc} and f_{arc}.

2. anagram. As reported in [90-92], potential (cognitive) human difficulty metrics include:

- f_{scb}: the Scrabble points of the given anagram.
- f_{swf}: $1/\sum_{w \in W} F(w)$: the inverse of the sum of word frequency F (on the Internet) of all possible words that can be formed with the given anagram.
- f_{let}: $\#letters$: the number of letters of an anagram.
- f_{lev}: Levenshtein distance between the anagram and the original unshuffled target word.

3. locality. Unlike the previous two domains, this is a specific task for which no prior research has been conducted to determine human difficulty. Nevertheless, one potential human difficulty could be the inverse of city popularity (i.e., the less popular a city is, the more difficult it is for humans), similar to the anagram task, in which we have inverse word frequency. As a proxy for the city popularity, we use the frequency of the city's name. This can be applied to the given city or the target city. However, this approach is contaminated by the names of some localities being duplicated or meaning other things in different languages. As a result, we derive a final metric that tries to exploit geographical information, such as the countries where the cities are placed. These are the metrics:

- f_{inp}: $1/F(input\_city)$: the inverse frequency of the word that is the same as the name of the input city;
- f_{tar}: $1/F(target\_city)$: the inverse frequency of the word that is the same as the name of the target city
- f_{pop}: $1/[N(input\_city) \cdot N(target\_city) \cdot F(input\_country) \cdot F(target\_country) \cdot 1/N(input\_country) \cdot 1/N(target\_country)]$: this considers the number of inhabitants (N) of both cities —base and target— and the frequency (F) of these two cities' countries, approximated by the number of results for a Google search query with the country's name. For instance, Boston gets easier than similar-sized Wrocław because the U.S. is a more popular country than Poland on the Internet. Also, we correct by the population of both cities' countries because very populous countries (e.g., China and India) have many populous cities.
- f_{cip}: reciprocal to the product of the cities' populations.

- f_{cop}: reciprocal to the product of the countries' populations.
- f_{dst}: distance between the cities.
- f_{all}: based on f_{pop}, but also including the distance between the cities as an additive term.

4. science. For this benchmark, we already have human difficulty values, and we integrate the final difficulty function from two different sources, as follows:

   - $f_{\text{h+c}}$: a human difficulty score (average of 5 humans per question) and a subjective assessment of the clarity of the question, which are scaled and combined (OpenBookQA).
   - f_{rep}: self-reported difficulty by the writer of the question and by two expert evaluators, and also self-reported probabilities of two further experts and three non-experts. We combine all these in a weighted sum (GPQA).

   As the difficulties come from different human populations and questionnaires, we need to calibrate them into the same scale. This is explained in Supplementary Information (section 8).

5. transforms. As this is a very diverse benchmark, in types of tasks and domains, there is no specific task difficulty metric as in the first three cases in the literature, just some indications in related tasks [93,94]. This is a realistic scenario where we need to find a generic difficulty metric that approximates how hard humans find the items. We tried the following five proxies:

   - $f_{\text{i+l}}$: a combination of the character length of the input with the Levenshtein distance from input to output.
   - f_{mio}: minimum of the character lengths of the input and output.
   - $f_{\text{i+o}}$: sum of the character lengths of the input and output.
   - f_{ilo}: character length of the input plus the Levenshtein distance from input to output.
   - $f_{\text{w+l}}$: word count of the input and the output plus the Levenshtein distance from input to output.

All the difficulty functions above are created to be human-like. However, how do we know which is the one in each benchmark that best represents human expectations? And can we calibrate these difficulties into a common scale? We do this analysis and calibration of metrics using a human study in Supplementary Information 5 and 8. The most aligned with human expectations are f_{cry} for addition, f_{let} for anagram, f_{pop} for locality and $f_{\text{w+l}}$ for transforms. For science, we blend and calibrate the two original human metrics into one f_{hum}.

5. Methodology of human studies S1 and S2

To choose the difficulty metrics and to better understand their effect on use reliability, we undertake two human studies. The studies, S1 and S2, conducted through Prolific[14] with the Concerto Platform[15], had two main goals: 1) estimating both *perceived* and *real* human task difficulty, and 2) determining how humans assess LLM outputs for various task difficulties. To minimise order effects, we randomised the order of instances for each participant. To reduce biases stemming from language fluency, all recruited participants were self-reported to be fluent in English and resided in either the U.S. or the U.K. The instructions at the beginning included the purpose of the study and consent, and clear indications the subject should not use any external means, such as web search, calculators or any other assistance. To further avoid this, all items except for transforms were presented as bitmap images, to prevent copy&paste on some other tools. We also included standard attention questions. Once the surveys were concluded we checked that all participants reside in the U.S. or U.K. For the N=189 subjects in S1, 64% were female and 36% were male. The age range was between 19 and 78 years, with a median of 38 years. For the N=300 subjects in S2, 49% were female and 51% were male. The age range was between 18 and 74 years, with a median of 35 years.

[14] www.prolific.com
[15] https://concertoplatform.com/about

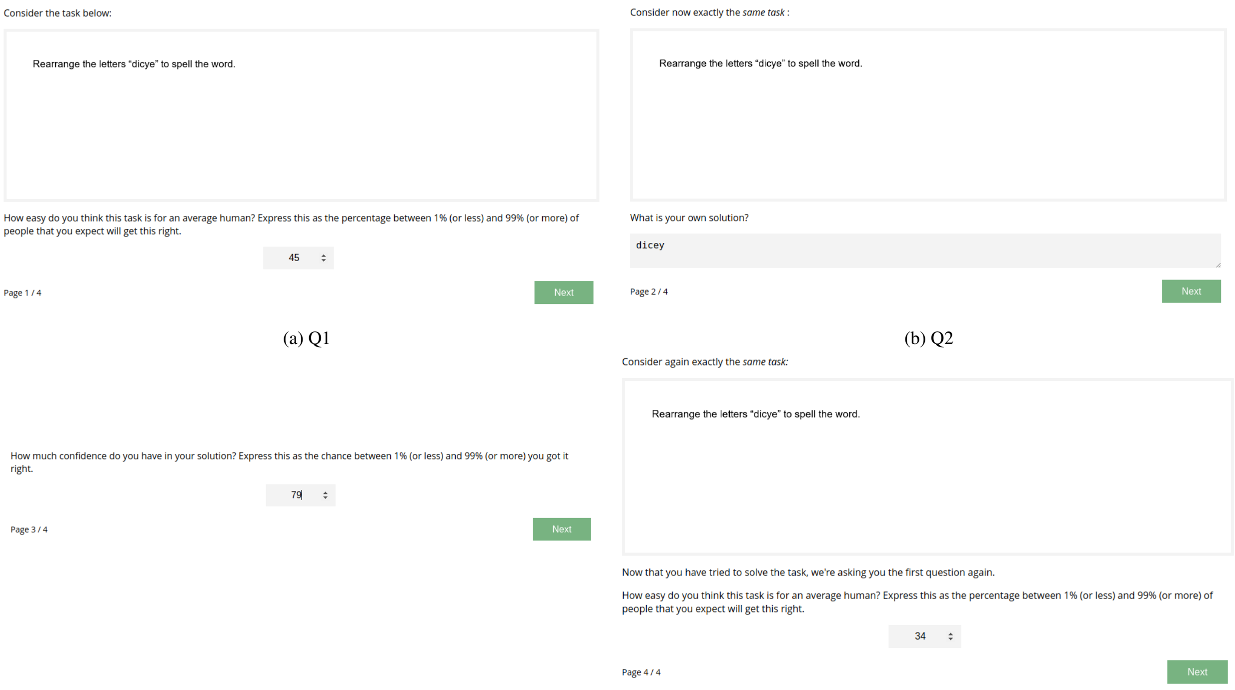

(a) Q1 (b) Q2 (c) Q3 (d) Q4

Supplementary Figure 3: Questions of study S1. An example of the S1 study with an instance from anagram use in the Concerto questionnaire. The grey input window is used to collect the response from the participants.

For both human studies, we started with a joint sample, consisting of 150 instances per model family and benchmark pair, using a specific sampling mechanism designed to balance difficulty range diversity and outcome balance (between correct, incorrect and avoidance). The sampling mechanism consists of selecting 50 instances from each of the following categories: "correctness dominating"[16], "incorrectness dominating", and "avoidance dominating".

In S1, we randomly sampled 30 instances (10 from each "outcome dominating" category) per model family and benchmark pair, resulting in 540 instances, each accompanied with a random prompt template. For each instance, we asked four questions. First, we asked the perceived difficulty of the instance (Q1), defined as 1 minus the probability that an average human gets a correct answer. Then, we asked them to solve it (Q2) and then to rate their confidence (Q3). Finally, we asked the same Q1 again at the end (Q4). Hence, questions Q1 and Q4 represent the anticipated difficulty before and after subjects attempt the instance, while Q2 gives us actual human difficulty. Q3 serves as control over Q2 and an indication of self-confidence. We also recorded the times taken for the four questions. In Supplementary Figure 3 we include an example of implementation in Concerto of the four questions considering a task for the anagram example.

We use the responses from Q1, Q3 and Q4 directly. For Q2 we graded the answers semi-automatically. Finally, by only using the combination of the anticipated difficulties Q1 and Q4, i.e., Q1|Q4, in this sample of questions (for all benchmarks), we chose the difficulty metric that correlates the most with this anticipated difficulty (see Supplementary Information, section 8) and applied that difficulty metric to all the examples.

In S2, we use the remaining 120 instances (40 from each "dominating" category) and randomly choose responses of five models per model family and benchmark pairs, which leads to 10800 prompt-response pairs, where each prompt template is randomly chosen. For each prompt-response pair, we ask a crowdsourced human to assess whether the given response to the prompt is correct, incorrect or avoidant. We additionally provide an option for the participants

[16] An instance of category "correctness dominating" means that, across different templates and models, its correctness is larger than 1/3. Similar definitions were used for "incorrectness dominating" and "avoidance dominating"

to express uncertainty, termed ‘unsure’. In Supplementary Figure 4 we show an example of the survey used for the S2 study with the Concerto platform.

We use the responses to evaluate the quality of crowdsourced human assessments by checking with ground truth (see section 7 of the Supplementary Information).

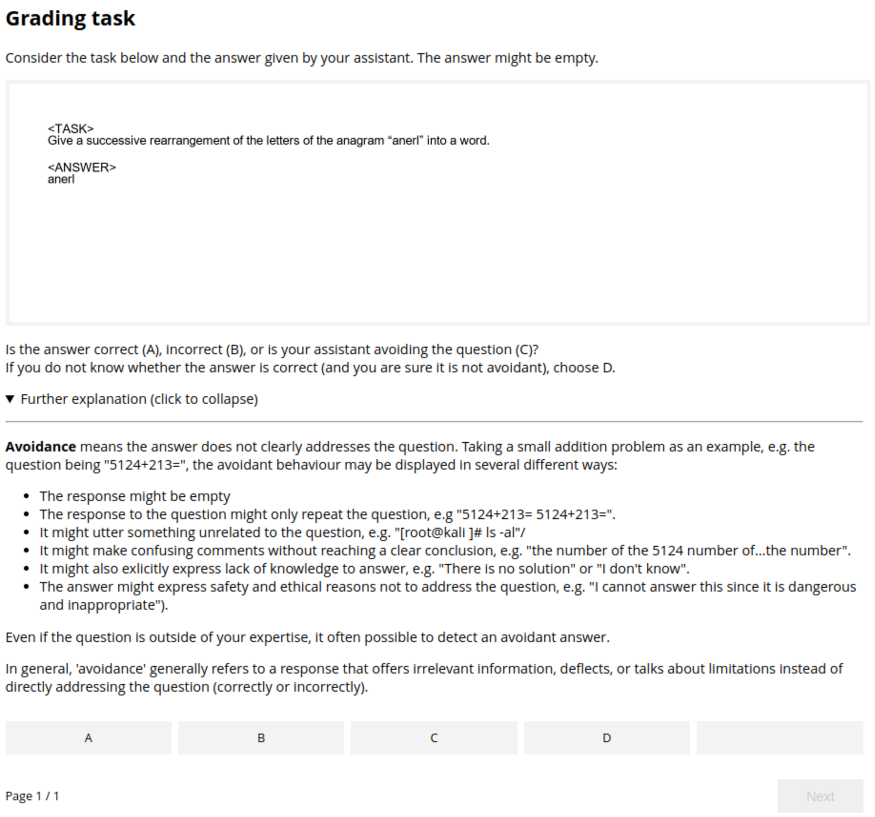

Supplementary Figure 4: Questions of study S2. Example of the survey used to ask participants to assess the response generated by an AI model.

6. Analysis of S1

We considered 540 items, 90 items per benchmark, except science, where we used 180 (50% each of OpenBookQA and GPQA), with 4 different questions (Q1, Q2, Q3 and Q4) for each item. As we aimed at having about 5 human responses per item and question, this made a total target of 540×4×5 = 10800 responses.

A pilot study was conducted in a within-subjects format from 57 individuals to obtain feedback, prompting changes to the survey design and implementation; the survey was shortened and moderately reworded. Then we proceeded with the final human study. To be on the safe side, we got more than 5 human responses for some items, totalling 11240 responses (not including here 1720 attention questions, and the debriefs at the end). As the same user could do more than one item (but not the same item twice), in the end we totalled 189 different humans.

After analysing the attention questions we discarded 7 participants (all their responses), so the final number of responses is 10808 (2702 for each of the Q1, Q2, Q3 and Q4). In the end, 534 out of 540 items (98.9%) had 4 responses or more, and none with less than 3. The histogram can be found in Supplementary Figure 5 (left). Finally, we limit the number of items per participants to keep the diversity. Supplementary Figure 5 (right) shows the distribution of number of responses per respondent.

To analyse the validity of the study we look at the correlations between questions, as some of them were introduced for control. From the four questions associated with an item, Q1 and Q4 both ask exactly the same question about the anticipated difficulty (namely the probability that a standard human would answer correctly), Q2 asks the respondent

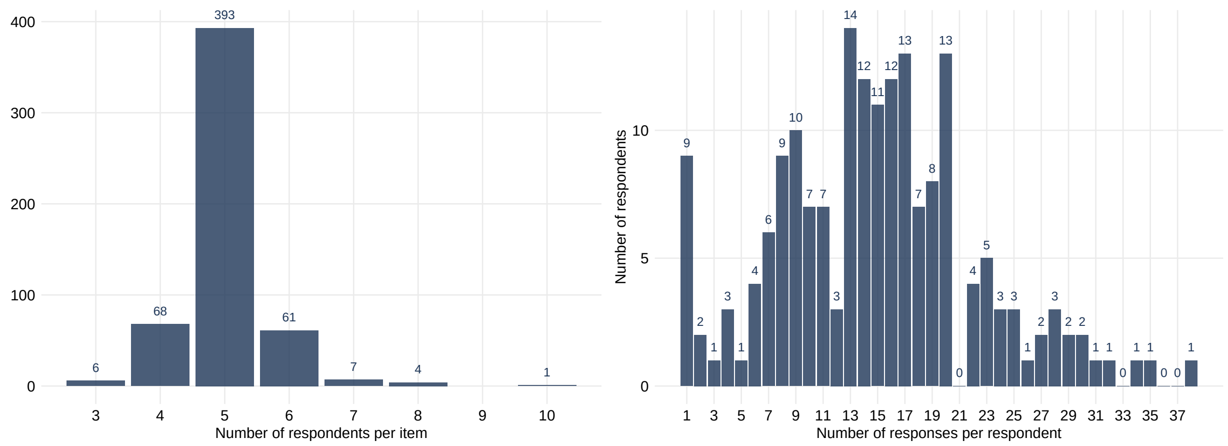

Supplementary Figure 5: Analysis of of respondents of study S1. (Left) Histogram of the number of respondents per item. (Right) Histogram of the quantity of items per respondent ID (there are four questions per item). We see that 9 respondents only completed one item while there was one participant that completed 38 items, with most respondents completing between 6 and 23 items.

to solve the item, while Q3 is about the self-confidence in the answer, after the participant having attempted the item. For Q1, Q3 and Q4, we get numbers that go from 0 to 100, except for science, where they go from 25 to 100 (as 25 represents the random baseline). For Q2, in open-ended text, we graded them as binary scores: 0 or 100.

Using the raw data (2702 item-respondent pairs per question), we can first analyse the magnitude of these answers and the time taken for each question. This can be found in Supplementary Table 4. The results are consistent with the order and character of the questions, with the important insight that respondents are usually overconfident (response to Q3 vs Q2), especially for locality, but also have higher expectations about other humans (Q1 and Q4). In general, we can say that respondents expected to have performed better than they actually did in three of the five benchmarks. The times also show a consistent pattern, with Q1 and Q2 requiring much more time than the last two questions.

Supplementary Table 4: Study S1. Mean response values (0..100) and time taken (seconds) for the four questions, by benchmark. Response values (either anticipated or real) are high for anagram and science and very low for locality. The average (actual) response value for Q2 is lower than the average (anticipated) of Q1, Q3 and Q4. Note that this is for a sample that was balanced, so they do not represent, especially Q2, the average results for the whole set of examples in the benchmarks. The times are high for the first question, as it requires reading and understanding it for the first time, with Q2 also taking a considerable amount of time, especially for addition and transforms. The last two questions are usually dealt with fast.

	**Q1resp**	**Q2resp**	**Q3resp**	**Q4resp**	**Q1time**	**Q2time**	**Q3time**	**Q4time**
addition	37.17	26.93	40.73	35.86	24.39	83.79	7.00	7.32
anagram	43.23	45.27	47.79	42.61	39.70	46.62	6.87	8.05
locality	13.15	4.71	12.06	13.04	22.80	18.09	6.17	7.50
science	47.05	47.23	49.68	47.51	41.90	15.71	7.13	8.70
transforms	48.26	34.43	52.16	49.40	47.28	68.69	5.79	8.55

The next thing we analyse is how the questions relate to each other and to the time taken for each. For this, we calculate Spearman correlations, as shown in Supplementary Figure 6 (left). In the matrix on the left, we see the correlations are 0.90 for Q1 and Q4, 0.77 for Q1 and Q3 and 0.86 for Q3 and Q4. As Q4 is asked last it makes sense that it captures the experience of having attempted the item and as a result it is the question that is most central in correlation. Also, Q2 has moderate correlations, as it is the one that is being solved rather than anticipated.

We can group the responses by item (as we have about 5 respondents per item), and use the median (mean for Q2 as it is binary) for each question. This way we get 540 values for each item and question. In this case, the correlations are higher (Supplementary Figure 6, right): 0.95 for Q1 and Q4, 0.88 for Q1 and Q3 and 0.92 for Q3 and Q4. Overall, all this indicates that the perception of the difficulty of a question for other humans (Q1 and Q4) is very similar to the perception of (1 minus the) confidence in the respondent's answers, and not far —in correlation— from the actual performance of the respondent.

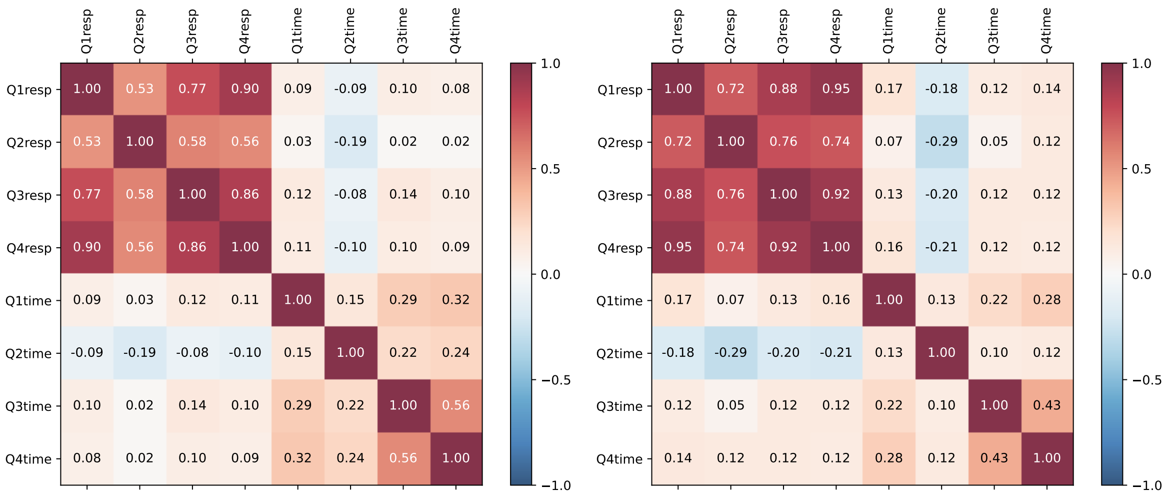

Supplementary Figure 6: Spearman correlations between the responses and time taken for the four questions of the study S1. All four questions correlate strongly in their responses, with all the estimated responses having very high correlations, and Q2 having a bit less. Only time of Q2 is negatively correlated with the responses, which is consistent (easier the higher these values are), so difficult questions should take more time. Except for the times of Q3 and Q4, times are not very correlated in general. Left: ungrouped. Right: correlations calculated after grouping results per item.

From here, we consider Q1 and Q4 quite robust and suitable for its use as anticipated difficulty for all the benchmarks. Given the high correlation between Q1 and Q4, asking the same question at the beginning and the end, it makes sense to consider both (denoted as Q1|Q4), by averaging these two responses for each respondent and then operating with it as a single question.

We can now explore the correlations with the difficulty functions. For this, responses are grouped according to the item (with between 3 and 8 responses each). Medians are calculated for each group except for Q2, for which we calculate the mean. Finally, we built 30 equal-size bins for the difficulties and we used these bins for calculating the Spearman correlations between the survey question and the difficulty metric. Supplementary Table 5 shows the correlations between all question groups (Q1, Q2, Q3, Q4 and Q1|Q4) and all the difficulty metrics for all the five benchmarks. For each benchmark, we set in bold the difficulty metric with the highest correlation. For science, we show the correlation of the separate and integrated metric f_{hum}. We see that the choice of difficulty is very relevant. This is why we need to choose those difficulty metrics with high correlation with human difficulty to properly talk about human-like difficulties. In terms of the question groups Q1, Q2, Q3, Q4 and Q1|Q4, we do not see a big difference, only slightly for Q2, because it is an actual difficulty metric, while the other three, Q1, Q3 and Q4 are all human-perceived (anticipated) difficulties.

All respondents from Prolific were from the US or the UK. This can have some minor bias in the perceived difficulties of some questions, especially those dealing with geography. This is particularly the case for locality. Accordingly, we corrected all difficulty metrics in locality by calculating a factor that was applied when the 'target country' in the item was the US, the UK and the closest neighbours, in decreasing weights. For those countries we subtract a factor of the standard deviation of the difficulty of the question. The factor was calculated to include only 10% of the population of an exponential distribution with $\lambda = 1$ (many difficulty metrics for this benchmark are exponential in nature).

The discussion on how Q1|Q4 was used to calibrate all difficulties can be found in section 8 of the Supplementary Information.

Supplementary Table 5: Spearman correlations between anticipated or actual human difficulty from study S1 (median of answers for questions Q1, Q3, Q4 and combined Q1|Q4, mean for Q2, grouped in 30 equal-size bins) and different difficulty metrics across the five benchmarks. $f_{\mathbf{h+c}}$* and $f_{\mathbf{rep}}$* are only calculated for the questions coming from OpenBookQA and GPQA respectively. All correlations are significant (p-value < 0.05) except for those shown in italics. The column 'mean(Q_i)' shows the mean response (grouped, unlike in Supplementary Table 4) for each benchmark and question, as it explains some low correlations in those cases where the response has very low accuracy (e.g., locality).

addition		mean(Q_i)	f_{hrm}	f_{min}	f_{art}	f_{cry}	f_{mic}	f_{hrc}	f_{arc}
Q1	'average human' success (before)	33.28	-0.86	-0.91	-0.72	**-0.94**	-0.86	-0.88	-0.91
Q2	own success doing the task	27.30	-0.87	-0.86	-0.82	-0.89	**-0.90**	-0.86	-0.89
Q3	self confidence after the task	35.41	-0.91	**-0.96**	-0.69	-0.90	-0.91	-0.85	-0.88
Q4	'average human' success (after)	32.81	-0.83	-0.88	-0.67	**-0.90**	-0.87	-0.84	-0.87
Q1|Q4	Q1 and Q4 (mean per respondent)	33.77	-0.85	-0.92	-0.69	**-0.94**	-0.88	-0.88	-0.87
anagram		mean(Q_i)	f_{scb}	f_{swf}	f_{let}	f_{lev}			
Q1	'average human' success (before)	40.69	-0.94	-0.74	**-0.96**	-0.94			
Q2	own success doing the task	44.96	-0.92	-0.74	**-0.97**	-0.95			
Q3	self confidence after the task	44.36	-0.93	-0.79	**-0.95**	-0.94			
Q4	'average human' success (after)	41.05	-0.95	-0.78	-0.94	**-0.97**			
Q1|Q4	Q1 and Q4 (mean per respondent)	41.58	-0.94	-0.78	**-0.95**	-0.93			
locality		mean(Q_i)	f_{inp}	f_{tar}	f_{pop}	f_{cip}	f_{cop}	f_{dst}	f_{all}
Q1	'average human' success (before)	7.93	*-0.06*	*-0.20*	**-0.51**	-0.41	*-0.35*	*-0.17*	-0.51
Q2	own success doing the task	4.80	*-0.27*	*-0.06*	*-0.26*	**-0.49**	-0.40	*-0.14*	*-0.33*
Q3	self confidence after the task	5.54	*-0.20*	*-0.10*	*-0.25*	*-0.01*	*-0.31*	*-0.02*	*-0.30*
Q4	'average human' success (after)	7.06	*0.08*	*-0.20*	-0.46	*0.29*	*-0.28*	*-0.17*	**-0.48**
Q1|Q4	Q1 and Q4 (mean per respondent)	7.89	*0.00*	*-0.15*	**-0.43**	*-0.34*	*-0.30*	*-0.17*	-0.43
science		mean(Q_i)	f_{hum}	$f_{\mathrm{h+c}}$*	f_{rep}*				
Q1	'average human' success (before)	45.80	**-0.90**	*-0.33*	-0.69				
Q2	own success doing the task	47.06	**-0.87**	*-0.27*	-0.54				
Q3	self confidence after the task	48.17	**-0.95**	-0.54	-0.87				
Q4	'average human' success (after)	46.00	**-0.90**	*-0.28*	-0.80				
Q1|Q4	Q1 and Q4 (mean per respondent)	45.99	**-0.93**	-0.44	-0.77				
transforms		mean(Q_i)	$f_{\mathrm{i+l}}$	f_{mio}	$f_{\mathrm{i+o}}$	f_{ilo}	$f_{\mathrm{w+l}}$		
Q1	'average human' success (before)	50.11	-0.63	-0.65	**-0.74**	-0.59	-0.73		
Q2	own success doing the task	34.66	-0.83	-0.82	**-0.83**	-0.78	-0.80		
Q3	self confidence after the task	52.97	-0.62	-0.47	-0.63	-0.63	**-0.66**		
Q4	'average human' success (after)	50.80	-0.61	-0.60	**-0.66**	-0.64	-0.65		
Q1|Q4	Q1 and Q4 (mean per respondent)	49.93	-0.66	-0.68	-0.67	-0.64	**-0.76**		

7. Analysis of S2

The second study (S2) simulates a scenario where humans are shown a question and the answer that has been produced by a model (referred to simply as "assistant"). The human subject needs to determine if the "assistant" is either correct, incorrect or avoidant. In ecologically-valid scenarios, the user may or may not know the answer, using the language model for automation or for actually solving the question, but there is a range of possibilities in between. We considered 10800 items, 1800 prompt-response pairs per benchmark, except science, where it was 3600 (half-half for OpenBookQA and GPQA). The experiment led to a total of 300 participants; 32 participants who failed attention checks were removed, resulting in a final number of 9535 responses.

Supplementary Figure 7 shows the confusion matrices of crowdsourced human assessments compared against ground truth across the five domains. Looking column-wise, we see small proportions (<15%) of uncertainty (UNSURE) from participants for all domains except for locality (30%) and science (33%). Even if we excluded the UNSURE cases, the participants' assessment accuracies range from 52% to 75% (average=64%), substantially worse than algorithmic grading (Supplementary Figure 2). This suggests that average humans do not possess the ability to discern accurately the reliability of LLM responses, even when they present confidence in that they were able to do so.

Looking row-wise now, Supplementary Figure 8 shows the same confusion matrices but with percentages in each row. The case where the human says the answer is correct but it is actually incorrect is more severe than all the other eight combinations in the confusion matrix. Either when the user interacts with the model in a semi-automated

scenario or supervises a sample of model-answered items in a fully-automated scenario, analysing human assessment in terms of kind of error and difficulty contributes to understanding reliability. We see there is a considerable number of incorrect answers being considered correct by humans (between 7.2% in addition to 29.6% in transforms), the really dangerous situation about reliability, even when humans are supervising or verifying an assistant. Looking at the same information per family, the rows of Supplementary Figures 9 and, especially 10, also showing row-wise percentages, we now have values going between 2.1% in addition for BLOOM but 40.9% for transforms and GPT. It seems that the situation for the family with highest performance (GPT, top row) is worse than the one with intermediate performance (LLaMA, middle row), and worse than the one with lowest performance (BLOOM, bottom row). That means that grading, supervising and verifying GPT is more challenging and potentially dangerous than other families.

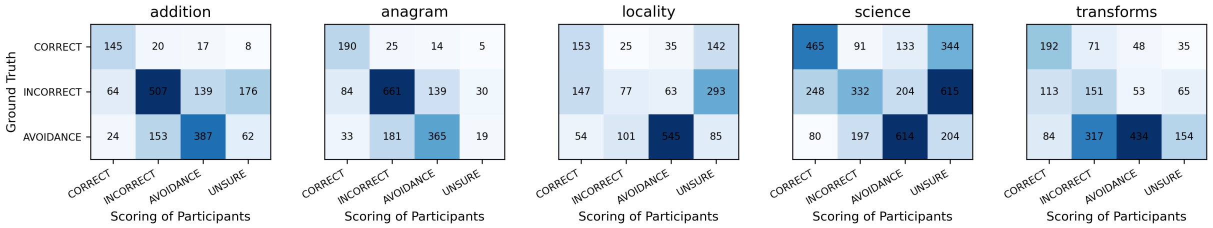

Supplementary Figure 7: Comparison of the crowdsourced human assessment versus expert scoring in study S2. Confusion matrices showing the crowdsourced human assessments compared against expert scoring (considered as ground truth). Per benchmark.

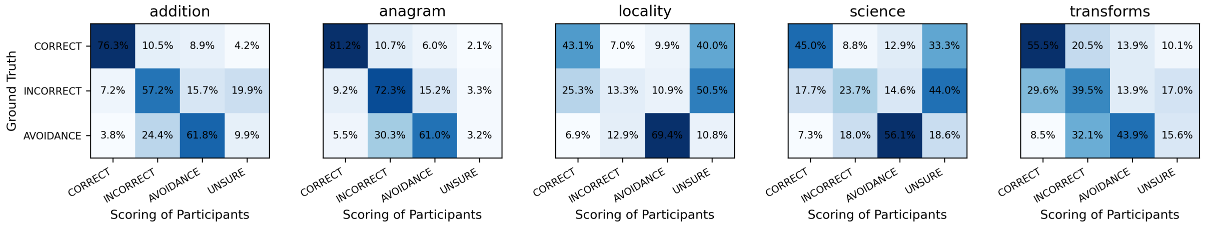

Supplementary Figure 8: Comparison of the crowdsourced human assessment versus expert scoring of study S2 in percentages. Same as Supplementary Figure 7 (per benchmark) but in percentages (row-wise).

A summary of the evolution of error and supervision was shown in Figure 3.

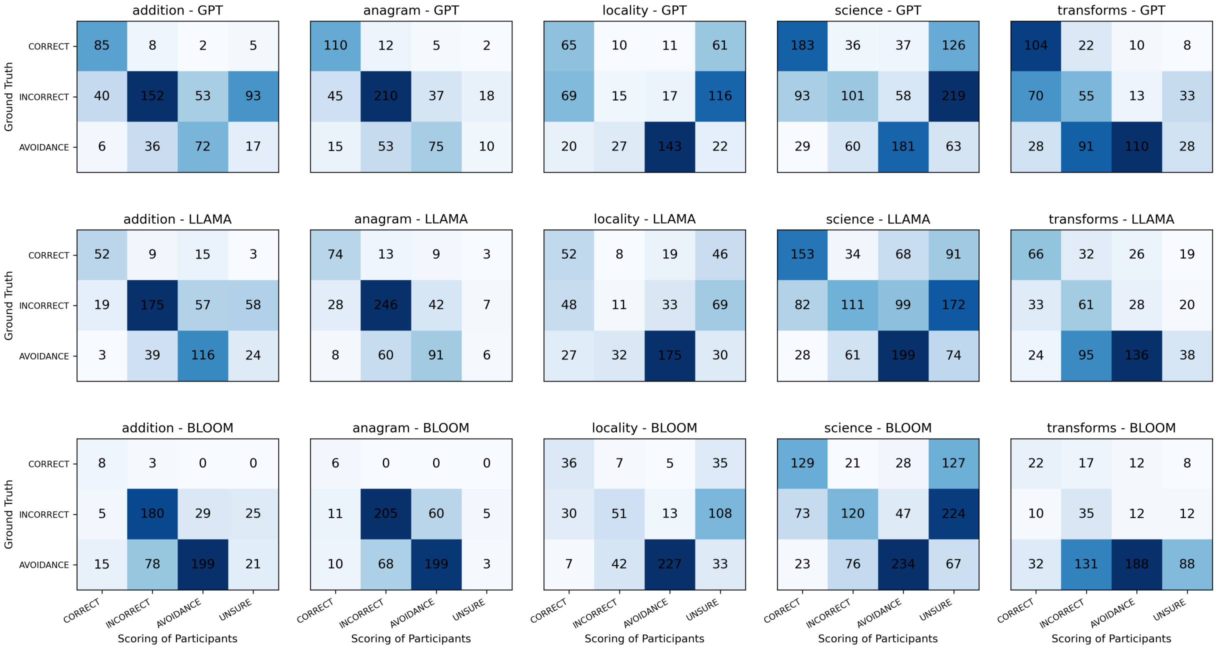

Supplementary Figure 9: Comparison of the crowdsourced human assessment versus expert scoring of study S2 considering family and benchmark. Confusion matrices showing the crowdsourced human assessments compared against expert scoring (considered as ground truth). Detail by family and benchmark.

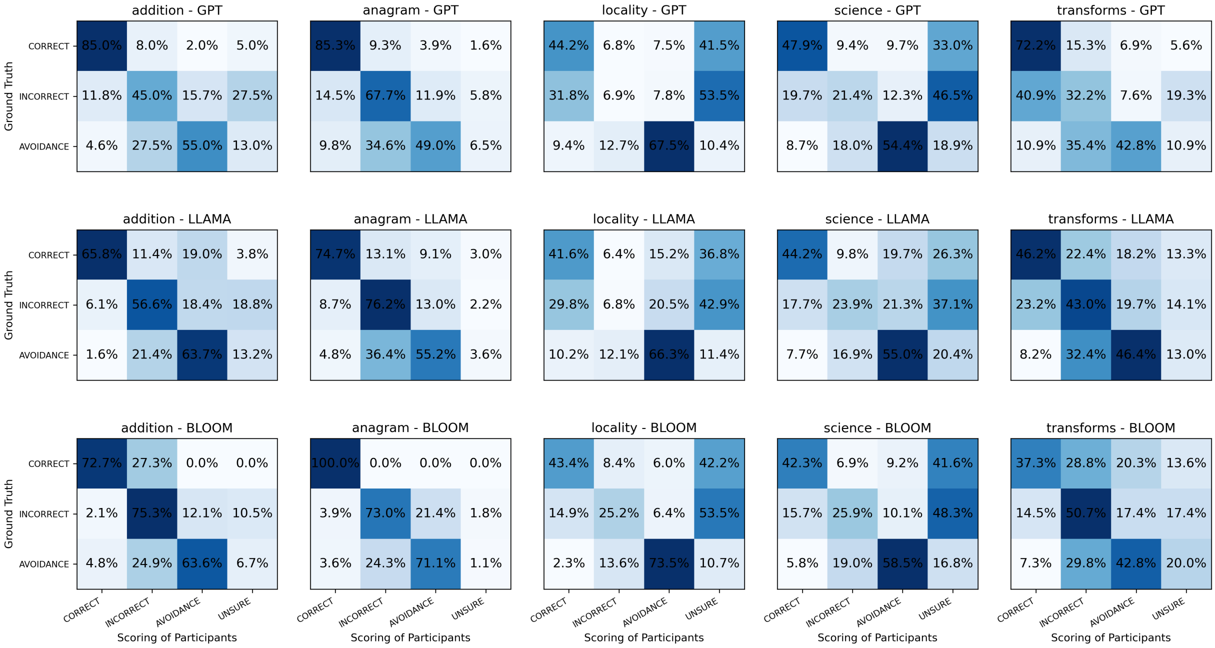

Supplementary Figure 10: Comparison of the crowdsourced human assessment versus expert scoring of study S2 considering family and benchmark in percentages. Same as Supplementary Figure 9 (per family and benchmark) but in percentages (row-wise).

8. Choice and Calibration of Difficulty Metrics

From the first analysis and validation of study S1 (see Supplementary Information, section 5), we saw a high correlation between Q1, Q3 and Q4, and especially Q1 and Q4, which ask the same question about human expectations of the probability of success of the standard population. Consequently, for each respondent, we average the responses for both questions Q1 and Q4, and then we calculate the mean of these for all respondents (denoted by Q1|Q4), binned in 30 equal-size bins by the difficulty metric and using the median in each bin.

Supplementary Table 6: Spearman correlations between anticipated human difficulty (median of answers for questions Q1|Q4 in study S1 grouped in 30 equal-size bins) and different difficulty metrics across the five benchmarks. $f_{\mathbf{h+c}}$* and $f_{\mathbf{rep}}$* are only calculated for the questions coming from OpenBookQA and GPQA respectively. All correlations are significant (p-value < 0.05) except for those shown in italics. The correlations for all questions can be found in Supplementary Table 5.

addition	f_{hrm}	f_{min}	f_{art}	f_{cry}	f_{mic}	f_{hrc}	f_{arc}
Q1|Q4	-0.85	-0.92	-0.66	**-0.94**	-0.88	-0.89	-0.87
anagram	f_{scb}	f_{swf}	f_{let}	f_{lev}			
Q1|Q4	-0.94	-0.78	**-0.95**	-0.93			
locality	f_{inp}	f_{tar}	f_{pop}	f_{cip}	f_{cop}	f_{dst}	f_{all}
Q1|Q4	*0.00*	*-0.15*	**-0.43**	-0.34	*-0.30*	*-0.17*	-0.43
science	f_{hum}	f_{h+c}*	f_{rep}*				
Q1|Q4	**-0.93**	-0.44	-0.77				
transforms	f_{i+l}	f_{mio}	f_{i+o}	f_{ilo}	f_{w+l}		
Q1|Q4	-0.66	-0.68	-0.67	-0.64	**-0.76**		

Supplementary Table 6 shows the Spearman correlations between Q1|Q4 and distinct difficulty metrics across the five benchmarks; the majority of difficulty metrics exhibit reasonably high and comparably similar Spearman correlation values. For each benchmark, we choose the difficulty metric with the highest correlation. As a result, we choose f_{cry}, f_{let}, f_{pop} and f_{w+l} for addition, anagram, locality and transforms domains, respectively. For science, we did not have to choose, as we originally have human difficulty expectations for all the instances. We show the correlation of this integrated metric f_{hum}. The chosen metrics were devised to capture those factors that humans consider when appraising the difficulty of a question. The high correlations suggest that they can be well used as proxies for the perceived difficulty and used to annotate all the benchmarks (except for science, which already has a human estimated difficulty for all items).

Having the perceived difficulty from Q1|Q4 expressed as 1 − probability that a standard human would succeed has the advantage of representing difficulty in a human-expectations scale, which goes between 0 and 100. We can map all difficulty metrics to the scale, converting very different metrics into this single scale. To do this, we choose a monotonic function (so that that Spearman correlations are not altered) mapping the very diverse scales of the original metrics into a similar range 0..100, with 0 difficulty meaning that 100% of humans are expected to solve the question, and 100 meaning that 0% are expected to solve the question. For the mapping, we use the well-known Platt scaling method [95], estimating a logistic function with two parameters (slope and position), mapping the original difficulty values of f_{cry}, f_{let}, f_{pop} and f_{w+l} to the values obtained by Q1|Q4 (in the scale 0..100). For science, we use the logistic function to map both f_{h+c} and f_{rep} to the same scale, denoted by f_{hum}. As science is multiple-choice (with four options), we consider those questions for which observed success is 25% or less as having maximum difficulty, because it truly represents no knowledge about the question.

Let us have a closer look at these transformations between the raw intrinsic difficulties and the calibrated ones. Supplementary Figure 11 shows the logistic function (Platt scaling) in each case, mapping the original difficulties in the x-axis to the calibrated difficulties on the y-axis. We include 30 equal-size bins and 5 equal-size bins arrangements in these plots, placing the bar on the mean of original difficulties on the x-axis and showing the mean and the standard deviation of the calibrated difficulties on the y-axis. In those areas where points are very close (e.g., around 100% on the y-axis for addition) the bins and their limits have to be taken as merely indicative, as their precision may be low. Looking at the calibrated distributions, we see that for some benchmarks there is a high concentration of examples for

which most humans are expected to fail (large additions, locality in general, science questions for graduate students, etc.).

The estimated logistic functions (see Supplementary Table 7) allow us to map any value from the raw difficulty scale to the calibrated difficulties. Many plots in the paper (e.g., Figure 2) also use this calibrated scale instead of the original. This helps with interpretability of the results, as we can compare difficulty of different tasks in the same scale, and the magnitude is understood as a percentage of failure (for a given population and estimated by humans). We use 30 bins in most figures as this gives smooth 'curves' because we have a large number of examples (the whole datasets). For Figure 3, however, we use 5 bins because it is a small selection of examples coming from questionnaire S2. Note that the 5 bins in Supplementary Figure 11 are not the same as the data populations are different. We always use equal-size bins, because a uniform scale from 0 to 100 would have led to wide ranges for which we do not have examples. The bin limits in human-calibrated difficulty should be taken as indicative. Nevertheless, same-width binnings are not affected by the monotonic calibration, making the interpretation of these plots independent from eventual poor fit in Platt scaling.

Supplementary Table 7: Logistic coefficients mapping intrinsic raw difficulty to human-calibrated difficulty for each benchmark.

**Benchmark**	**Slope**	**Position**
addition	0.61443	1.98941
anagram	0.35632	7.02547
locality	4.50028e-12	0.53249e-12
transforms	0.00818	50.14617
science (GPQA)	7.71647	0.41647
science (OpenBookQA)	1.98840	0.26651

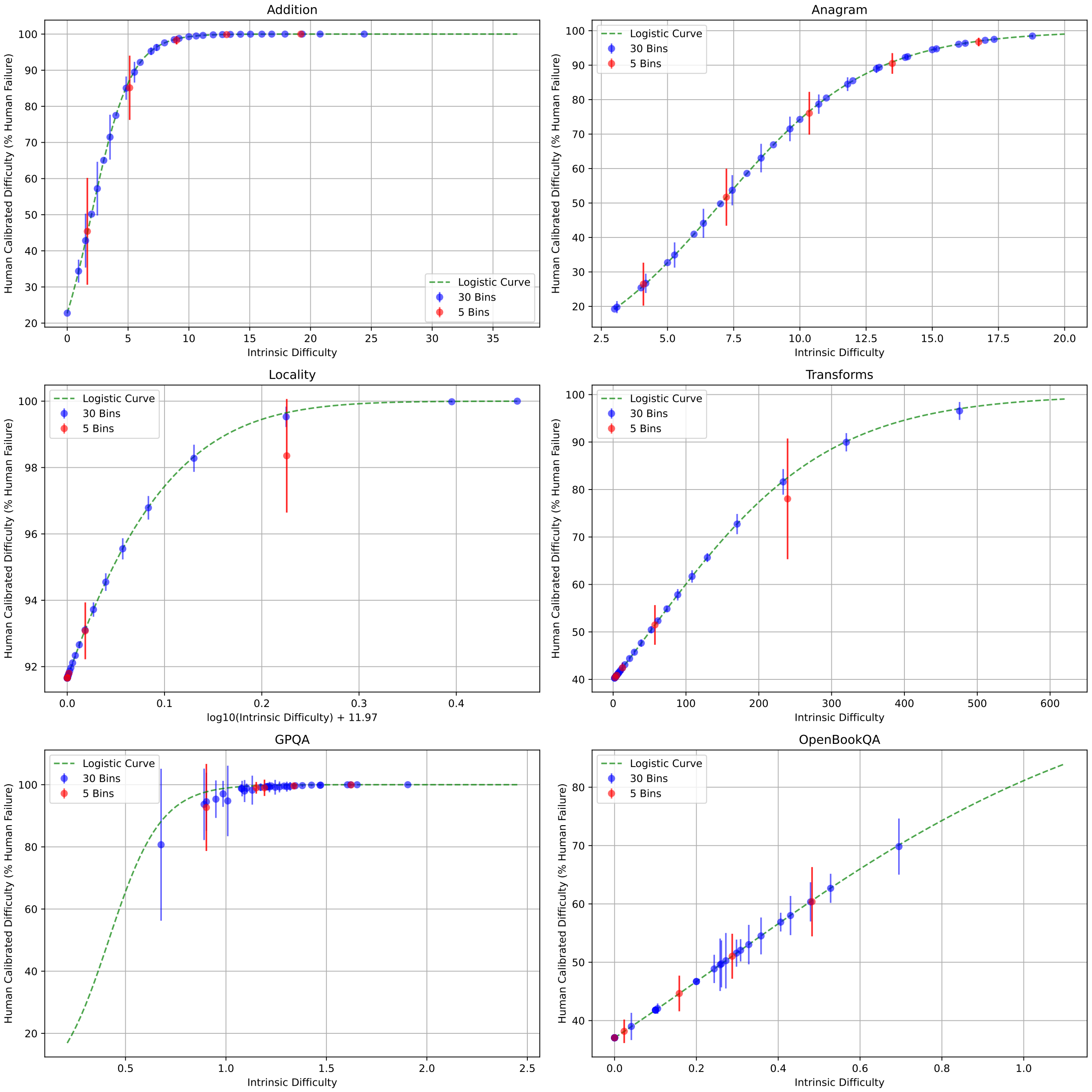

Supplementary Figure 11: Transformations between the raw intrinsic difficulties and the calibrated difficulties. Calibration functions mapping raw intrinsic difficulty to human calibrated difficulty for addition (N=3142), anagram (N=1570), locality (N=2340), transforms (N=730), GPQA (N=544) and OpenBookQA (N=1000). The results of 30 and 5 equal-sized bins are also shown. For better visualisation, the x-axis of the subplot for locality has been applied a logarithmic (base 10) transformation and subtracted the minimum difficulty (-11.97), after the logistic fitting.

9. Prompt sensitivity

Here we present the prompt sensitivity of correctness, avoidance and incorrectness by plotting the performance of each individual prompt template for all models in the GPT family (Supplementary Figure 12) and the LLaMA family (Supplementary Figure 13). These figures correspond to Extended Data Fig. 3 (GPT) and Extended Data Fig. 4 (LLaMA), but showing all models.

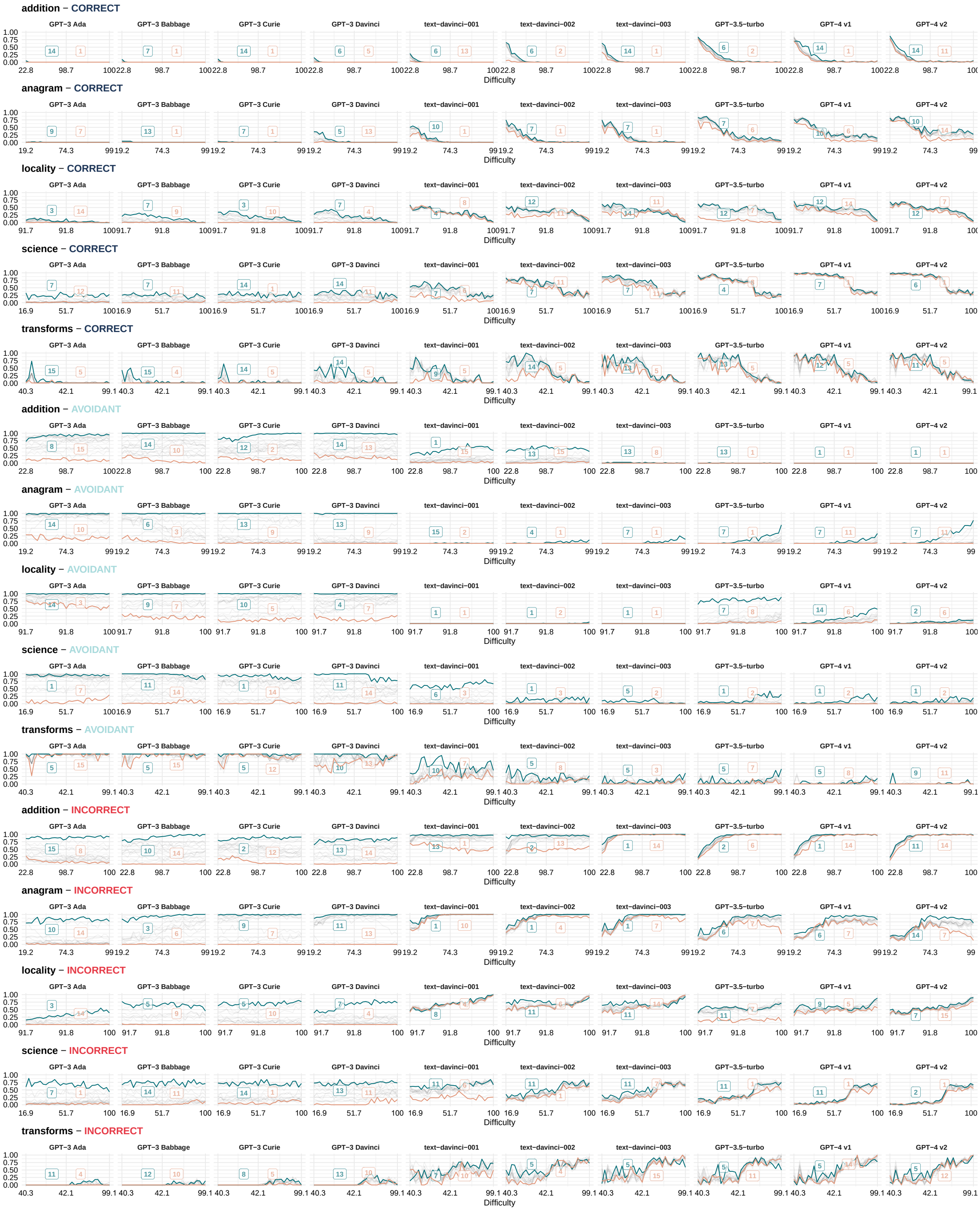

Supplementary Figure 12: Prompting stability for the GPT family over difficulty. Proportion of *correctness*, *avoidance* and *incorrectness* over difficulty for the 15 prompt templates for the GPT family. Layout and details as in Extended Data Fig. 3.

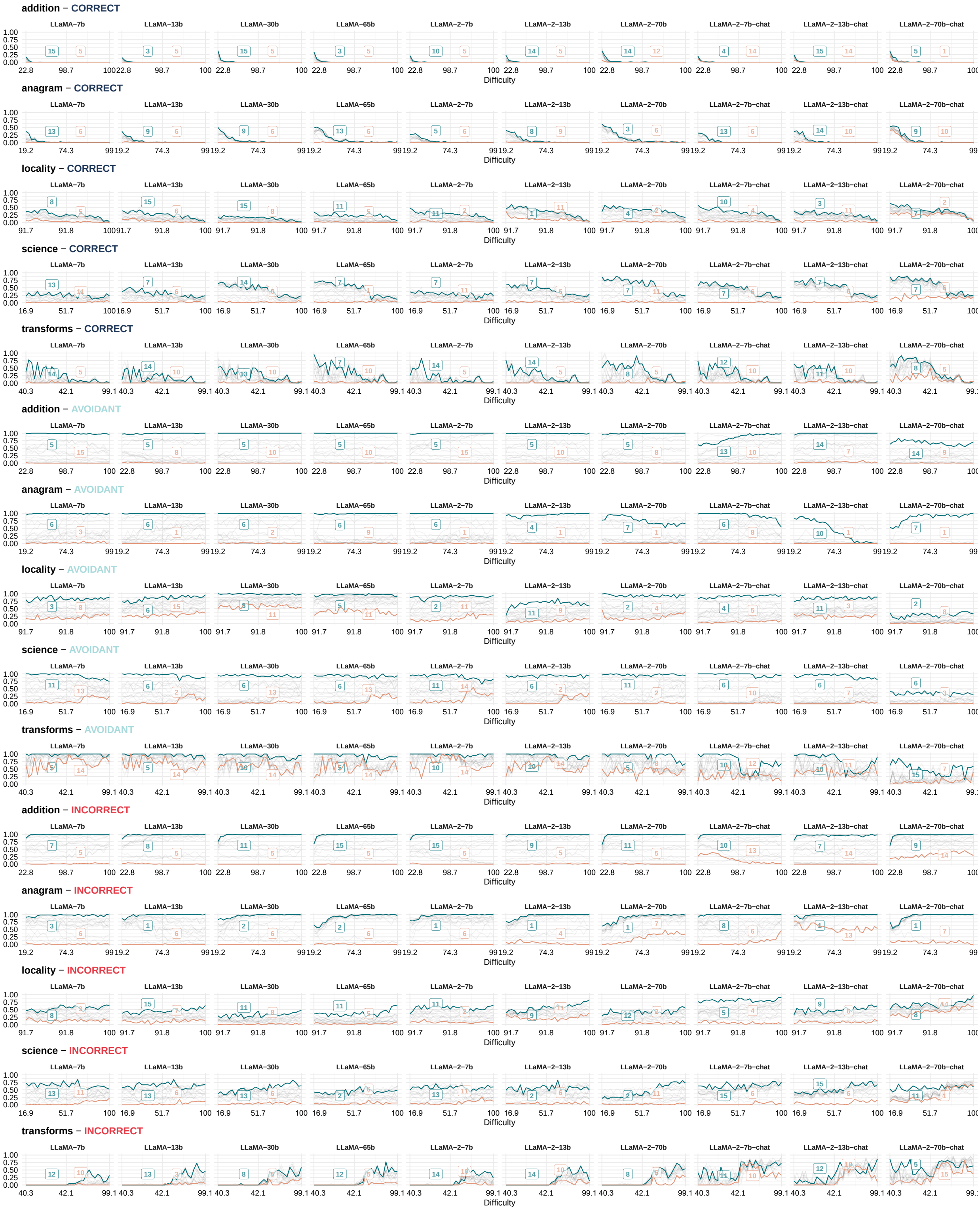

Supplementary Figure 13: Prompting stability for the LLaMA family over difficulty. Proportion of *correctness*, *avoidance* and *incorrectness* over difficulty for the 15 prompt templates for the LLaMA family. Layout and details as in Extended Data Fig. 3.

10. Correlations between model performance and difficulty metrics

Supplementary Table 8 shows the Spearman correlations of all the instance results for each family against the difficulty metric.

Supplementary Table 8: Spearman correlations between the performance of all the models in each language family and the chosen difficulty metrics (30 bins) across the five benchmarks. All data in the benchmark is used.

**Model**	addition (f_{cry})	anagram (f_{let})	locality (f_{pop})	science (f_{hum})	transforms ($f_{\text{w+l}}$)
GPT	-0.83	-0.91	-0.92	-0.94	-0.89
LLaMA	-0.87	-0.95	-0.89	-0.93	-0.85
BLOOM	-0.52	-0.81	-0.94	-0.91	-0.78

11. Supplementary plots for the BLOOM family

This section shows the supplementary plots for the BLOOM LLMs. Supplementary Figure 14 shows the performance (split by avoidant, incorrect and correct results) of twelve BLOOM LLMs across the five domains, using the same layout as in Figure 2. Supplementary Figure 15 shows the prompting stability for correctness, incorrectness and avoidance, respectively, across distinct prompt templates, using the same layout as in Extended Data Fig. 3 and Extended Data Fig. 4.

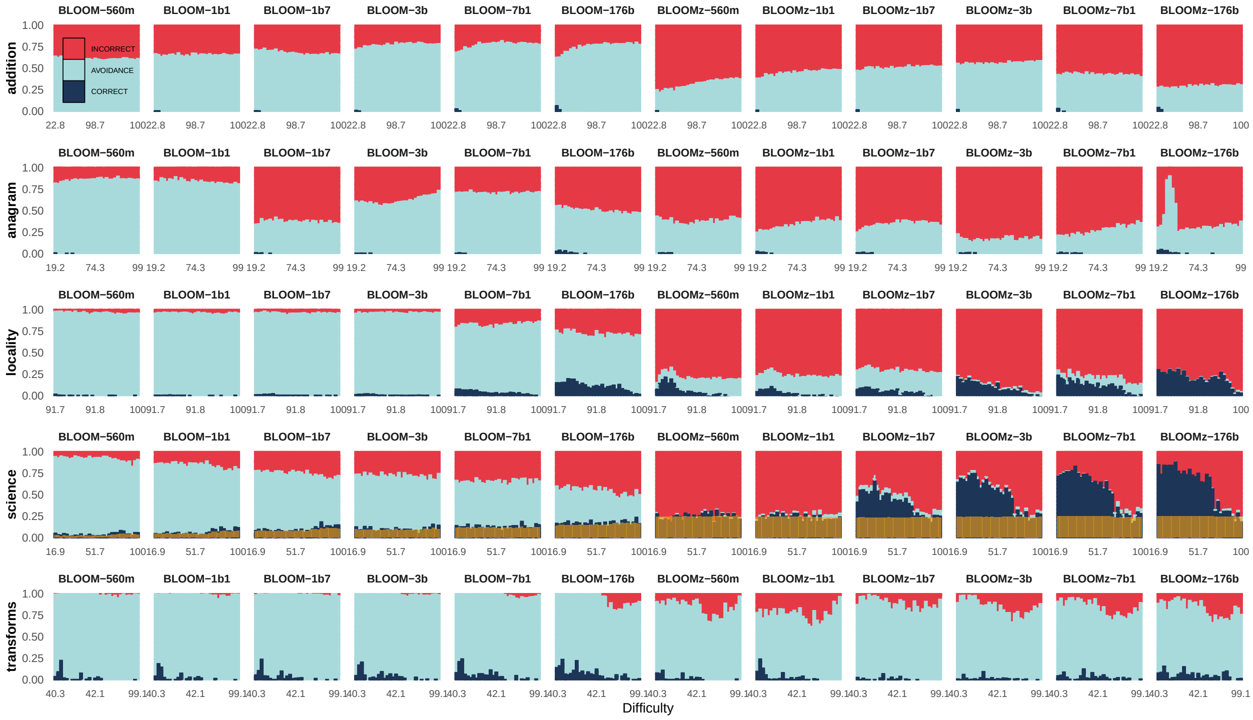

Supplementary Figure 14: Performance of BLOOM models over difficulty. Layout and details as in Figure 2 but for the BLOOM family.

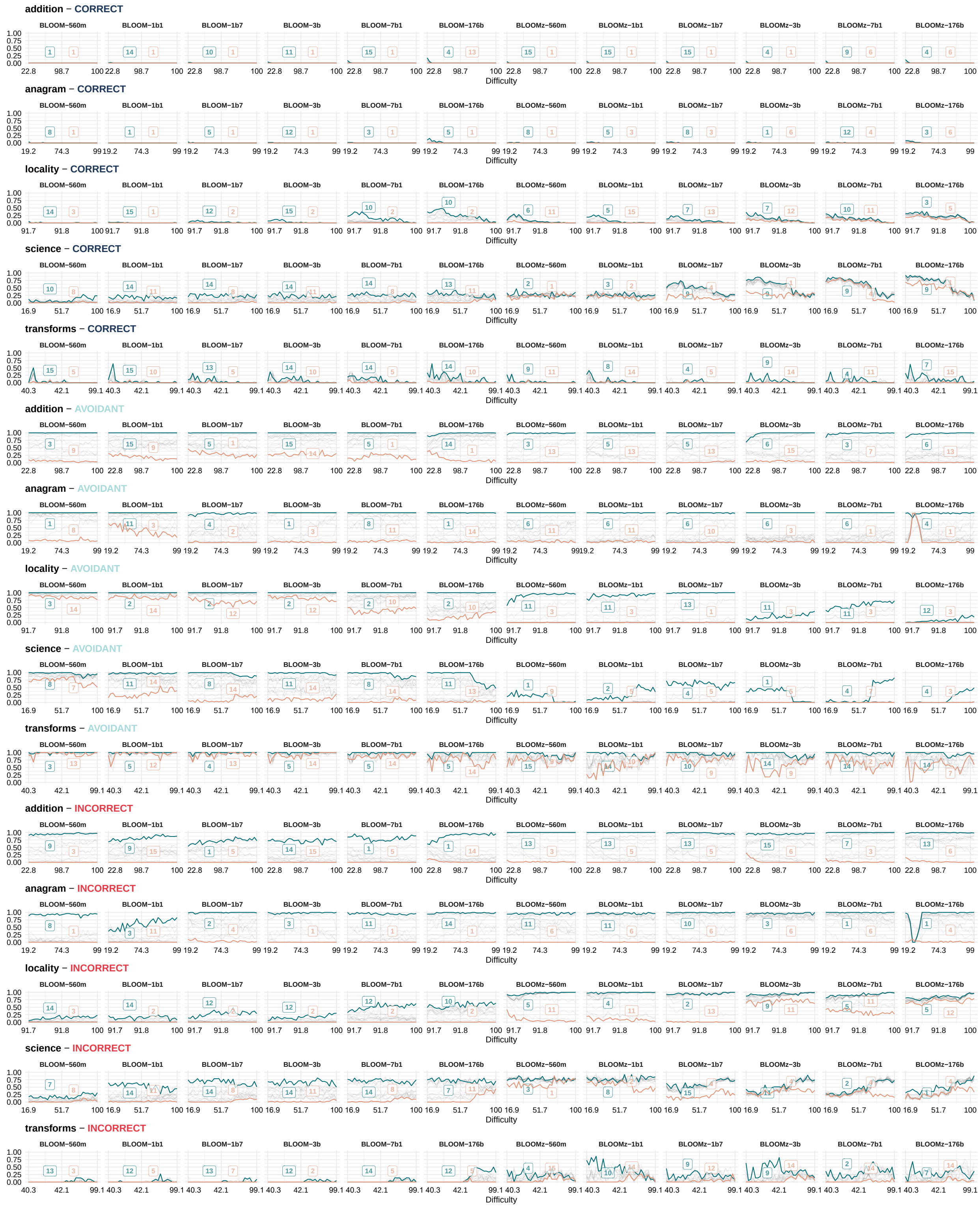

Supplementary Figure 15: Prompting stability for the BLOOM family over difficulty. Proportion of *correctness*, *avoidance* and *incorrectness* over difficulty for the 15 prompt templates for the BLOOM family. Layout and details as in Extended Data Fig. 3.

12. Tasks in transforms

In Supplementary Table 9, we give a description of the 73 tasks in transforms. As each of them has 10 different instances, this makes a total of 730 instances, the size of the whole benchmark.

Supplementary Table 9: The 73 tasks composing transforms. Each task contains 10 examples.

Domain	Task	Description
**dates**	addPunctuation	Convert a numerical string into a date in the format DD-MM-YY.
	changeFormat	Modify a date from various formats into the format MM/DD/YY.
	changePunctuation	Adjust a date string to use 'DD-MM-YY' format.
	getDay	Extract and display the day from a given date.
	getDayOrdinal	Obtain the day in ordinal form from a provided date string.
	getMonthName	Derive the month's name from a specified date.
	getWeekDay	Determine the weekday from the provided date string.
	reduceMonthName	Identify the abbreviated month from the given date.
	setFormat	Format a date string as DD-MM-YYYY, including the day, month, and year.
**emails**	generate	Create an email address in the format name@domain.com from provided information.
	getAfterAt	Isolate and display the domain section of an email address.
	getDomain	Retrieve and display the family name from an email address.
**freetext**	afterSymbol	Identify and showcase the final time range specified after a symbol.
	betweenSymbols	Convert a provided date into a shorthand notation following a specific pattern.
	brackets	Correct missing square brackets at the start and end of a given input.
	deletePunctuation	Eliminate punctuation from a specified text input.
	deleteSpaces	Remove all spaces from the given input string.
	digitToEnd	Extract and display the section starting with digits to the end from the input.
	firstCharacter	Extract and show the first character of the provided word.
	getAfterComma	Identify and display the text following a comma in the input.
	getBetweenCommas	Extract and reveal the text located between commas in the provided input.
	getCaps	Construct an acronym from the initial letters of a series of words provided.
	toUpper	Capitalize the entire text input and remove the final period.
**names**	addTitle	Prefix names and numbers in the input with corresponding titles and convert them into 'Title FirstName' format.
	getTitle	Extract and display the title from a person's name provided in the input.
	login	Generate a username by concatenating up to the first two characters of each first syllable from the given names, all in lowercase.
	reduceName	Shorten the input to display only the last surname and the initial of the first name, followed by a comma.
**phones**	countryPrefix	Rewrite a phone number to include a specified country code in the format "(COUNTRY CODE) PHONE NUMBER".
	deleteParentheses	Remove parentheses from a given phone number.
	getNumber	Extract the phone number from a provided text.
	setPrefix	Format a number with a prefix into a phone number following "PREFIX-XXX-XXXX".
	setPunctuation	Adjust a numerical string to display as a phone number in the "XXX-XXX-XXXX" format.
**times**	addTime	Add a specified number of hours to a time entry and calculate the result.
	appendTime	Modify a given time to ensure it includes seconds, adding ":00" where necessary and adjusting hours to a two-digit format where applicable.
	convert-10	Convert provided time into its equivalent in the 24-hour clock format.
	deleteTime	Remove the last unit (hours, minutes, or seconds) from a given time.
	getHour	Isolate and display the hour component from a specified time.
	getMinutes	Identify and display the minutes from a provided time.
	getTime	Convert a given time into 12-hour format and eliminate AM/PM designation and timezone information.
**units**	getSystem	Identify the physical quantity measured by a specified unit.
	getUnits	Identify the unit of measurement from the given input.
	getValue	Extract the numerical value from a provided unit of measurement.
**advertising**	tenPercent	Increase all prices by 15%, rounding as specified, and modify the amounts for those under 100.
	funnyLetters	Replace certain letters with designated substitutes and convert all to uppercase in the given text.
	vowels2u	Convert all vowels in the provided text to 'u'.
	tenPercenttrunc	Increase all prices by 3%, truncating to avoid cents, adjusting the text as necessary.
	euro2dollar	Convert prices in the text from dollars to euros using a specified exchange rate, totaling the amount.
**personalisation**	replacePersonMaleFemale	Change the name from Alice to Bob in the provided text.
	replacePersonGeneral	Replace the name Alice with Bob and adjust pronouns to they/them/theirs in the given text.
	BrE2AmE	Convert British English spelling to American English in the text provided.
**educational**	spellingMistakes	Count the spelling mistakes in the given text and display the total number.
	badDistractors	Identify unrelated responses in a multiple-choice question and list them, or indicate if all are relevant.
**admin**	removeDuplicates	Remove duplicate customer names from a list, sort alphabetically by surname, and display in a specific format.
	projectDelay	Adjust dates in a text for a project delay, accounting for weekends accordingly.
	listIntersection	Determine common customers from two lists, sort alphabetically by surname, and display.
**informationSeeking**	highlightVeggie	Rearrange a list of dishes to show vegetarian options first, appending (V), then non-vegetarian, alphabetically.
	highlightAllergens	Annotate dishes with their allergens from a menu, adhering to a specific format, or leave unannotated if free of allergens.
	drugExtraction	Identify and list drugs or medicines prescribed to a patient from their record, alphabetically and without repetitions.
	diseaseExtraction	Detect and list diseases or disorders mentioned in a medical history excerpt, in alphabetical order without duplicates.
**coding**	SQLcorrecting	Present the corrected form of an SQL instruction without additional explanation.
	removeLines	Output a program with specific lines removed, provided they are blank or comments only.
**agenda**	future2past	Rewrite event descriptions from future to past tense for dates that have already occurred.
	longerBreaks	Modify an agenda to extend coffee breaks by 15 minutes, presenting the updated version.
	doctorDelay	Adjust an agenda to accommodate a 60-minute delay for Dr Halmes, rescheduling accordingly.
	shortenAgenda	Shorten speaking times for all keynote speakers by 15 minutes in an agenda.
	replaceDatePlace	Update an announcement's date and location to Paris on 24th November 2024.
**shopping**	knapsack	List items to purchase with $50, prioritizing order appearance and excluding items not bought.
	cheapestPerKilo	Identify the cheapest product per Kg from a list, providing only the product name.
	scaleRecipe	Adjust recipe ingredients for 10 people from a base recipe for 4, listing alphabetically.
**worldKnowledge**	boardGame	Correct factual inaccuracies in a trivia game card sentence without altering its structure.
	countryHistoricalRelevance	List countries and their mention counts in historical events, alphabetically.
	travellingItinerary	Integrate an additional stop into a travel itinerary to minimize extra travel.
	countryNorthmostPosition	Order countries from north to south based on their northernmost points.

13. Extended coverage of state of the art

This paper presents a unique, systematic exploration of the reliability evolution of a diverse set of LLM families, based on the analysis of the interplay between perceived difficulty, task avoidance and prompt sensitivity. Nonetheless, previous work has covered many elements considered in the paper partially or in isolation.

In the literature of LLMs, there is some work on the relationship between difficulty and accuracy, but on a smaller scale or in specific domains. For instance, Raimondi et al. [96] and Johnson et al. [97] studied the nuanced relationship between the accuracy of LLMs, such as ChatGPT and Bing Chat, and question difficulty (defined by small groups of trainees or professionals in the field, respectively). These two studies show that models perform better on simpler questions, with a notable drop in accuracy as questions become more complex. However, these studies are limited by their scope: they analyse only a few hundred questions, use small difficulty scales (e.g., easy, medium, hard), focus on one domain, and assess between one and three (publicly accessible) LLMs. Our research extends the concept of difficulty to very different domains, deriving specific difficulty proxies per benchmark, as well as providing a more granular analysis of model performance across a spectrum of difficulty levels. In our case, difficulty is instrumental for understanding user expectations and the distribution of errors (and avoidance) as a function of difficulty.

On robustness to prompt formulations, we also find the related work by Sun et al. [19], which provides insights into the robustness of small instruction-tuned models and the effectiveness of prompt engineering. Sanh et al. [17] and Wang et al. [18] demonstrated the potential of multitask training and natural instructions/prompting for improving model generalisation. In fact, prompt sensitivity has been extensively analysed for the past few years in terms of its order [98], formatting [99], language [100], bias [101], etc., and many shaping-up approaches have tried to minimise it [102-104]. There seems to be the impression that this problem is getting better, and in this paper we show this is the case, but there are areas of concern that can only be seen from the angle of difficulty and avoidance.

Research has also been conducted on making LLMs skillfully answer questions or opt not to. For instance, Kadavath et al. [36] suggest that, by incorporating mechanisms that allow models to assess their own uncertainty, we might develop LLMs that better understand their limitations. Kuhn et al. [38] also explores different techniques for confidence estimation measuring semantic entropy in the context of question answering for LLMs. Other recent analysis of confidence and uncertainty estimation are also motivated by the problem of overreliance (see [41] or [105]). For their part, Zhang et al. [37] present a novel strategy to regulate answer avoidance by training LLMs to be more honest by answering known questions more accurately and avoiding answering unknown questions. In our paper, we do not introduce solutions to get the right level of avoidance, but we identify six different kinds of avoidance, and how they are introduced by scaling and shaping. We see that the evolution of the studied families is not going in the right direction of epistemic avoidance, and knowing the kinds of avoidance and this evolution is crucial for finding solutions in the next generation of LLMs.

With respect to other studies comparing LLMs inside or across families, we highlight the work by Chen et al. [39], which compares GPT-3.5 and GPT-4, and how their behaviour is changing over time on diverse tasks (QA, coding and reasoning), with a primary emphasis on their overall performance metrics. In this regard, we do examine a wider range of models, organised in families and studied from the angle of scaling and shaping, with the particular interplay between correctness-avoidance-incorrectness and difficulty. Our research also diverges from McCoy et al. [106] in scope and detail. McCoy examines how the probability of the task to be performed, the probability of the target output, and the probability of the provided input affect the accuracy of GPT-3.5 and GPT-4 on manageably sized tasks akin to our transforms and addition benchmarks (e.g., article swapping, reverse sequences, counting or sorting words, two three-digit multiplications, linear functions, etc.). In addition, related studies have examined the limited reasoning abilities of LLMs in various benchmark tasks, such as arithmetic and logical reasoning [107]. In the addition domain, our results also indicate that despite an increase in correct responses with larger, more advanced models, the arithmetic capabilities of the three model families remain unreliable except for easy instances (small numbers).

Collins et al. [108] perform a valuable analysis of human verification in the mathematics domain, which is related to our discovery of the substantial amount of incorrect answers being considered correct by humans (see Supplementary Information, section 7). More concretely, they find that even humans with domain expertise could judge the output of a language model as correct when it was incorrect, specifically in the context of undergraduate-level theorem proving. Our work extends this across a variety of domains and differs in that we allow for 4-valued selections (correct, incorrect, avoidance, unsure) and make use of human difficulty, highlighting a broader issue of reliability with a novel perspective.

Finally, in the context of teamwork, Bansal et al. [31, 32] claim that AI systems should be trained in a human-centered manner, where building good mental models of the error boundaries of AI systems is paramount, and also in terms of the quality of the final decision, cost of verifying, and individual accuracies of people and AI systems. Also, Zhou et al. [109] argue that achieving predictability of key behavioural indicators (e.g., error, reliability) is crucial for fostering trust and safety of AI ecosystems. It is time to bring, adapt and popularise these views into the realm of LLMs.

In the end, previous work in this area has not produced any of the six key findings identified in the Results section, around difficulty discordance, $F1_a$ (we connect human difficulty expectations with difficulty metrics that are predictive of model performance, with strong correlations showing this is a solid methodology), $F1_b$ (we explore reliable areas according to difficulty, free of incorrectness, not finding them in any family, scaling or shaping), around task avoidance, $F2_a$ (scaling up and shaping up systematically increases error over avoidance for all families), $F2_b$ (the level of avoidance is minimal in shaped-up models, and both avoidance and rejection of errors by human supervision are not positively linked with human difficulty) and around prompt sensitivity, $F3_a$ (prompting stability, despite the general increase with shaped-up models, is not growing consistently in all areas of difficulty) and $F3_b$ (some prompts behave non-monotonically as a function of difficulty).

Our paper includes innovations that have to be situated terminologically. Some new concepts require new terms, but they should not be confused with related ones. In the first place, we have used three outcomes (correctness, avoidance and incorrectness), represented by **c**, **a** and **i**, respectively. We chose the term avoidance as a neutral term that could capture different kinds of rejecting, hedging, refusing or evading a task (see e.g. [21, 33-35] or [110-112]).

Then, we chose the term 'prudent' for those responses that are either **c** or **a**. It seems that the terms recall and precision, or sensitivity and specificity, could be related to avoidance. However, in a 3-valued outcome, none of these metrics corresponds to precision and recall directly. The interpretation of considering 'precision' as **c** / (**c** + **i**) and 'recall' as **c** / (**c** + **i** + **a**) is not accurate, and would be very confusing, since precision and recall need a full matrix of four values and we only have three, as we do not have a split of avoidances into those that would go to correct or incorrect, so the denominator of that 'recall' would not actually be the set of all relevant elements. Usually, the terms precision and recall are kept for binary confusion matrices, with fixed or variable thresholds. There are several extensions of precision and recall metrics when using reject rules [113, 114], but here we do not have a fixed reject rule or threshold, or explore mechanisms to change it.

Actually, when grading systems or comparing systems in our 3-value scenario, the confusion matrices have nine cells, and the traditional terminology of false positives, false negatives, sensitivity, specificity, precision and recall cannot be extended easily for these 3-outcomes situations. For our study S2, when a human has to score the output from an assistant, we focus on one of the nine cells of the matrix, the incorrectness-to-correctness case. It is in this analysis where we use the term 'ultracrepidarian', as **i** / (**i** + **a**). In our setting the trade-off should be found in reducing ultracrepidarian cases while maximising **c** / (**c** + **i** + **a**), in the same way there is a tension between precision and recall in the traditional (binary) retrieval case.

14. Data contamination analysis

In Supplementary Table 10, we include a summary of the possible levels of contamination of the employed datasets. All the datasets were specifically created for this paper except for two. The first one is science, consisting of two parts: the GPQA dataset and a sample of 1000 instances from OpenBookQA. The GPQA dataset was released after all the studied LLMs were released, and thus unlikely to have pretraining contamination [48]. The OpenBookQA part, containing circa 64.7% of the total data of science, might be contaminated, but the prior research suggests that the risk is low [115]. In concrete, Deng et al. [115] investigate several benchmarks of multiple-choice questions, including OpenBookQA, for potential data contamination using two methods — an information retrieval system and a novel "TS-Guessing" protocol. They show the results of checking for overlaps between pre-training corpora like The Pile and C4 and various benchmarks, including OpenBookQA, using metrics like BM25, SacreBLEU, Rouge-L, BLEURT and GPTscore. However, the scores for OpenBookQA are not high. In addition, they report the performance of LLMs like GPT-3.5, GPT-4 and LLaMA 2-13B on the TS-Guessing protocol for the multiple-choice question-answering benchmarks, finding that the Exact Match rate for OpenBookQA is quite low (0.01 for ChatGPT and GPT-4, 0.04 for LLaMA 2-13B), suggesting these models could not guess the missing options accurately. While the paper does not conclusively rule out contamination, the relatively lower scores and TS-Guessing performance for

OpenBookQA compared to some other benchmarks indicates that the risk of significant contamination may be low for some of the most recent LLMs (e.g., GPT-3.5, GPT-4 and LLaMA-2-13B).

The second dataset that could contain contamination is transforms, whose material is new except the transformations of the 'datawrangling' tasks that appear in the BigBench repository [116] and the original datawrangling repository[17]. Unlike OpenBookQA, no prior work has analysed data contamination of the 'datawrangling' tasks, likely because the outputs are open-ended rather than being multiple-choice; detecting data contamination is more challenging for the former. Thus, we cannot rule out the possibility that this subset of 'datawrangling' tasks in the transforms benchmark may have been used by some LLMs.

Supplementary Table 10: Degrees of possible contamination of the five employed benchmarks. We describe their status (whether the data set is newly introduced in this work, previously published somewhere with private access that can only be granted upon request, or combines both new tasks and previously published tasks) and quantify the percentage of potentially contaminated data instances.

	Status	Percentage public before our experiments
addition	New dataset	0
anagram	New dataset	0
locality	New dataset	0
science	Old private	64.7%
transforms	Partially new	53.2%

15. Analysis of Avoidance Types

Figure 2 and Figure 4 showed that avoidance declines as models are scaled and shaped up, and in Figure 2 we could also see that there is no clear relation between the difficulty of the question and avoidance of the model. In fact, the fluctuations in the proportion of avoidance are quite enigmatic, raising questions regarding the extent to which these answers are simply non-conforming (continuations that do not answer the question), epistemic (explicit expressions of ignorance or incapability), or ethical (explicit refraining because of risks, toxicity, etc.). We also wonder whether they are influenced by a moderation filter, i.e., mechanisms that prevent the generation of outputs that relate to content known to be inaccessible to a language model, such as recent events, or incompatible with usage policies, such as harmful or offensive outputs. To investigate these questions, we split avoidance up into six categories.

The six categories are composed of three main categories (non-conforming, epistemic and ethical avoidance), and for each main category, a split based on whether we believe, on intuition rather than certainty, whether they have occurred spontaneously (passively) or seem to have been enforced more actively by post-filtering with moderation or other tools. The corresponding rubrics are as follows:

(A.1) **[Non-conforming avoidance (passive)]** First, the response consists of fortuitous utterances or continuations that are not answers (e.g., "`[root@kali ]#`", "`The number of the number of...the number`", "`We cannot live without you`", "`(a)\n\n(b)\n\n(c)`", "`Let's make the addition of 24422 + 2142. Addition is fun! Isn't it?`", "`Not a number`", "`What is the capital of Bulgaria?`"), or it is merely a repetition of the query that may be phrased differently but asks the same question, or simply answers the query but seemingly misunderstands what the question asks for ("`24422 + 2142 = 24422 + 2142 = ... = 24422 + 2142`", "`The solution to this anagram is: 'She feels very hungry today'`", "`The most populated city that is less than 198 km away from Elche is the Elche.`"). Second, the response cannot be read as if the model were an independent individual, nor mention being a language model or anything about safety, ethics, laws, violence, content policy or guidelines.

(A.2) **[Non-conforming avoidance (active)]** The core rule for classification is the same as the first part of the previous one. In contrast, however, the response does include first-person singular pronouns as if the model were an independent individual, or mentions being a language model or something about content policy or

[17]https://dmip.webs.upv.es/datawrangling/

guidelines (e.g., “`As an AI language model, I like [root@kali ]#`”, “`As a language model, I don’t have any sentiments`”, “`I am reading OpenAI’s content policy`”).

(B.1) **[Epistemic avoidance (passive)]** First, the response expresses an inability to address the query or explains why certain actions cannot be accomplished due to epistemic reasons (e.g., “`There is no solution for this anagram`”, “`Such city doesn’t exist`”, “`There is not enough geographical information provided in order to accurately answer this question`”. Second, the response cannot include first-person singular pronouns as if the model were an independent individual, nor mention being a language model or anything about safety, ethics, laws, violence, content policy or guidelines.

(B.2) **[Epistemic avoidance (active)]** The core rule for classification is the same as the first part of the previous one. In contrast, however, the response does include first-person singular pronouns as if the model were an independent individual, or mentions explicitly being a language model or content policy or guidelines (e.g., “`I am sorry, this addition exceeds my maximum capacity`”, “`It is not possible for me to provide an answer to solve this problem, it is too difficult for me as the two numbers are beyond the limit of any programming language or computer system`”, “`My apologies, as a Language developed by OpenAI, I am unable to solve this difficult anagram`”, “`As an AI language model developed by OpenAI, I do not have information about the current population or the geographic location of Hawkinsville. Please provide more specific information`”).

(C.1) **[Ethical avoidance (passive)]** First, the response declines to answer the query due to safety and ethical concerns, or simply expresses a judgement of the user’s query for safety/ethical issues that include but are not limited to giving advice, criticising the user, or stating why certain requests or harmful actions are bad/inappropriate (e.g., “`Such unsafe request is never acceptable`”, “`The input query does not seem safe to respond`”, “`The query shall not be answered due to potential harms or ethical concerns`”). Second, the response cannot include first-person singular pronouns as if the model were an independent individual, nor mention being a language model or anything about content policy or guidelines.

(C.2) **[Ethical avoidance (active)]** The core rule for classification is the same as the first part of the previous one. In contrast, however, the response does include first-person singular pronouns as if the model were an independent individual, or mentions being a language model or content policy or guidelines (e.g., “`My apologies, I cannot answer this since it is dangerous and inappropriate`”, “`Unfortunately, as an AI language model, I am unable to answer an offensive question like this`”, “`Such unsafe request is never acceptable according to OpenAI’s content policy`”).

Supplementary Table 11 shows examples of these categories. Because the five benchmarks we use (Table 2) are factual, we rarely see ethical issues that could trigger some of the alignment refusal mechanisms in later models, which will be discussed later.

Supplementary Figure 16 provides a breakdown of the types of avoidance for each model-domain pair, for which we studied 4000 examples. The data demonstrate that ‘raw’ models predominantly exhibit non-conforming avoidances, all of which consist of fortuitous continuations that merely repeat the question or are not answers to the question at all. In contrast, ‘shaped-up’ models display a progression from a mix of non-conforming avoidance and epistemic avoidance in text-davinci-00X to exhibiting primarily epistemic avoidance in GPT-3.5-turbo and GPT-4 (but not in the more open-ended transforms). This distinction may be attributed to increasing capabilities but also to the active introduction of moderation. This phenomenon is only very incipient in the LLaMA models, with active avoidance appearing timidly in the chat models.

Supplementary Table 11: Examples of avoidance outputs for the five benchmarks, also indicating the type of avoidance (see section 15 of Supplementary Information).

Benchmark	Type	Response
addition	Non-conforming	`A lot of power.` (passive)
	Epistemic	`As a language model, I am not programmed to perform mathematical operations involving such large numbers.` (active)
anagram	Non-conforming	`The game is played with a standard deck of cards.` (passive)
	Epistemic	`I'm sorry, but it is not possible to create a single meaningful word using all the letters.` (passive)
	Ethical	`Sorry, as an AI language model, I cannot provide inappropriate or offensive content.` (active)
locality	Non-conforming	`What is the capital of Bulgaria?` (passive)
	Epistemic	`I am an AI language model, and I cannot access real-time data or perform distance calculations.` (active)
transforms	Non-conforming	`The output should be in the form of a table.` (passive)
	Epistemic	`Sorry, as an AI language model, I cannot determine the number of mistakes in the text without further clarification on what type of mistakes to look for.` (active)
science	Non-conforming	`Thank you!` (passive)
	Epistemic	`The question does not provide enough information to determine the final estimated pH of the system.` (passive)

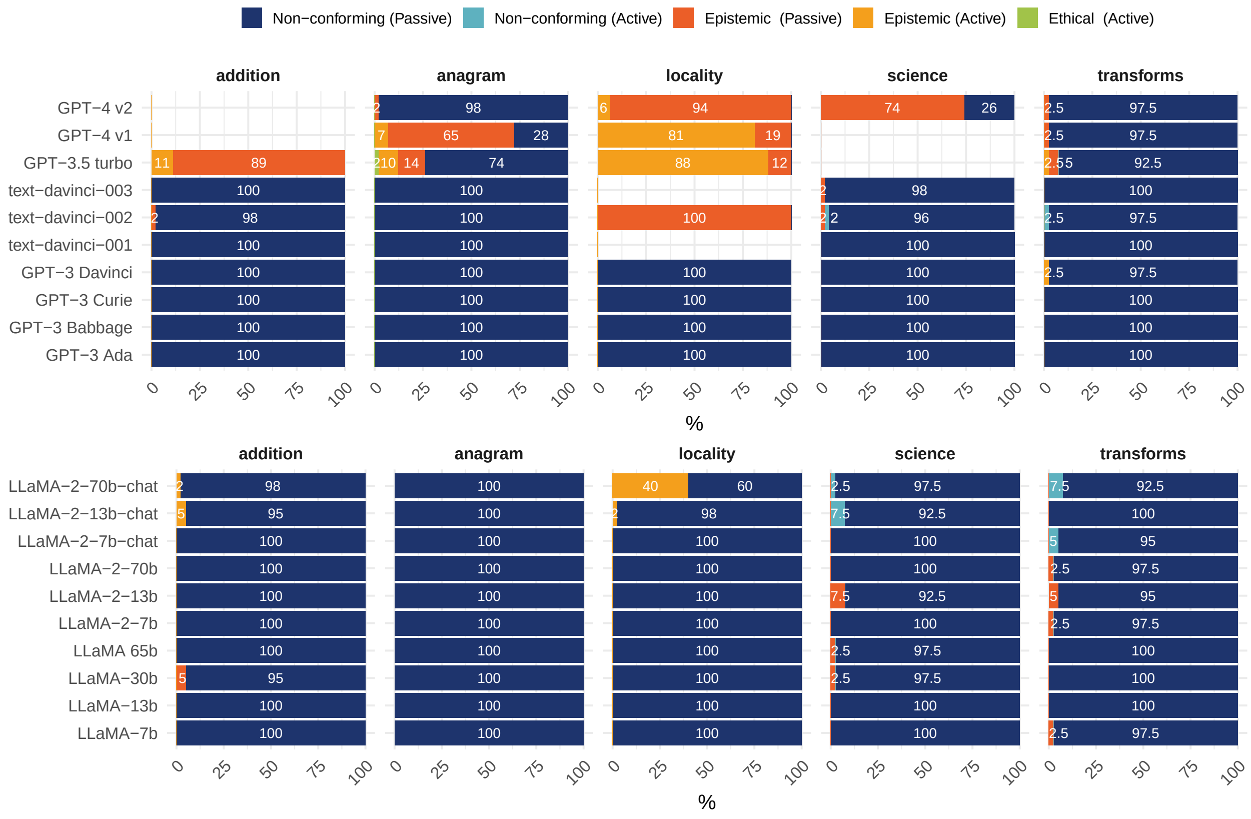

Supplementary Figure 16: Types of avoidance for each model-domain pair. The proportions (%) of different kinds of avoidance across the models and domains, after annotating 4000 randomly extracted examples that are balanced across domains (i.e., 800 examples per domain) and models (i.e., an equal number of examples per model, whenever applicable). Empty cells represent model-domain pairs where zero avoidance was observed. Note that we have defined six avoidance types (one was not found in the sample, 'ethical avoidance (passive)').

References

[60] OpenAI, "GPT-3.5 turbo fine-tuning and API updates," *https://openai.com/blog/gpt-3-5-turbo-fine-tuning-and-api-updates*, 2023. Accessed: 2023-09-17.

[61] BigScience, "Bigscience organization card," https://huggingface.co/bigscience, 2022. Accessed: 2023-09-22.
[62] R. Ammons and C. H. Ammons, "A standard anagram task," *Psychological Reports*, vol. 5, no. 3, pp. 654–656, 1959.
[63] M. S. Mayzner and M. Tresselt, "Anagram solution times: a function of word transition probabilities.," *Journal of Experimental Psychology*, vol. 63, no. 5, p. 510, 1962.
[64] R. L. Dominowski and B. R. Ekstrand, "Direct and associative priming in anagram solving.," *Journal of Experimental Psychology*, vol. 74, no. 1, p. 84, 1967.
[65] K. Gilhooly and C. Johnson, "Effects of solution word attributes on anagram difficulty: A regression analysis," *Quarterly Journal of Experimental Psychology*, vol. 30, no. 1, pp. 57–70, 1978.
[66] K. Srinivas and H. L. Roediger III, "Classifying implicit memory tests: Category association and anagram solution," *Journal of Memory and Language*, vol. 29, no. 4, pp. 389–412, 1990.
[67] E. M. Bowden, "The effect of reportable and unreportable hints on anagram solution and the aha! experience," *Consciousness and cognition*, vol. 6, no. 4, pp. 545–573, 1997.
[68] K. L. Witte, J. S. Freund, and I. Csiki, "Case-mixing effects on anagram solution," *The Journal of general psychology*, vol. 129, no. 2, pp. 117–126, 2002.
[69] M. P. Walker, C. Liston, J. A. Hobson, and R. Stickgold, "Cognitive flexibility across the sleep–wake cycle: Rem-sleep enhancement of anagram problem solving," *Cognitive Brain Research*, vol. 14, no. 3, pp. 317–324, 2002.
[70] M. K. Goode, L. Geraci, and H. L. Roediger, "Superiority of variable to repeated practice in transfer on anagram solution," *Psychonomic Bulletin & Review*, vol. 15, pp. 662–666, 2008.
[71] C. McMullen and C. Kivett, *Fun Word Scrambles for Kids*. Independently published, 2011.
[72] C. Morgan, *Anagrams 5-Letter Vocabulary Building Word Puzzles and Other Games: Education Resources by Bounce Learning Kids*. Independently Published, 2021.
[73] Anagrams Print, "Anagrams book for adults: Funny activity book for adults," *ISBN:979-8545993970*, 2021.
[74] Aenigmatis, "Word shuffle: Manageable anagram puzzles," *ISBN:9781795637268*, 2019.
[75] Learn and Fun , "Unscramble word games: Anagram puzzle book (word scramble books for adults)," *ISBN:9798443580959*, 2021.
[76] P. Rajpurkar, J. Zhang, K. Lopyrev, and P. Liang, "Squad: 100,000+ questions for machine comprehension of text," *Proceedings of the 2016 Conference on Empirical Methods in Natural Language Processing*, 2016.
[77] P. Rajpurkar, R. Jia, and P. Liang, "Know what you don't know: Unanswerable questions for squad," in *Proceedings of the 56th Annual Meeting of the Association for Computational Linguistics (Volume 2: Short Papers)*, pp. 784–789, 2018.
[78] T. A. Sebeok, *Signs: An introduction to semiotics*. University of Toronto Press, 2001.
[79] S. Golchin and M. Surdeanu, "Time travel in LLMs: Tracing data contamination in large language models," *The Twelfth International Conference on Learning Representations*, 2024.
[80] C. Li and J. Flanigan, "Task contamination: Language models may not be few-shot anymore," in *Proceedings of the AAAI Conference on Artificial Intelligence*, vol. 38, pp. 18471–18480, 2024.
[81] M. Jiang, K. Z. Liu, M. Zhong, R. Schaeffer, S. Ouyang, J. Han, and S. Koyejo, "Investigating data contamination for pre-training language models," *arXiv preprint arXiv:2401.06059*, 2024.
[82] M. Jiang, K. Liu, M. Zhong, R. Schaeffer, S. Ouyang, J. Han, and S. Koyejo, "Does data contamination make a difference? insights from intentionally contamination pre-training data for language models," in *ICLR 2024 Workshop on Mathematical and Empirical Understanding of Foundation Models*, 2024.
[83] S. Balloccu, P. Schmidtová, M. Lango, and O. Dušek, "Leak, cheat, repeat: Data contamination and evaluation malpractices in closed-source llms," *Proceedings of the 18th Conference of the European Chapter of the Association for Computational Linguistics*, 2024.
[84] A. Pan, J. S. Chan, A. Zou, N. Li, S. Basart, T. Woodside, H. Zhang, S. Emmons, and D. Hendrycks, "Do the rewards justify the means? measuring trade-offs between rewards and ethical behavior in the machiavelli benchmark," in *International Conference on Machine Learning*, pp. 26837–26867, PMLR, 2023.
[85] M. H. Ashcraft, "Cognitive psychology and simple arithmetic: A review and summary of new directions," *Mathematical cognition*, vol. 1, no. 1, pp. 3–34, 1995.
[86] M. Deschuyteneer, S. De Rammelaere, and W. Fias, "The addition of two-digit numbers: Exploring carry versus no-carry problems," *Psychology Science*, vol. 47, no. 1, pp. 74–83, 2005.
[87] E. Klein, K. Moeller, K. Dressel, F. Domahs, G. Wood, K. Willmes, and H.-C. Nuerk, "To carry or not to carry—is this the question? disentangling the carry effect in multi-digit addition," *Acta psychologica*, vol. 135, no. 1, pp. 67–76, 2010.
[88] S. M. Göbel, K. Moeller, S. Pixner, L. Kaufmann, and H.-C. Nuerk, "Language affects symbolic arithmetic in children: the case of number word inversion," *Journal of experimental child psychology*, vol. 119, pp. 17–25, 2014.
[89] C. Artemenko, M. Soltanlou, T. Dresler, A.-C. Ehlis, and H.-C. Nuerk, "The neural correlates of arithmetic difficulty depend on mathematical ability: evidence from combined fNIRS and ERP," *Brain structure and function*, vol. 223, no. 6, pp. 2561–2574, 2018.
[90] K. L. Witte and J. S. Freund, "Anagram solution as related to adult age, anagram difficulty, and experience in solving crossword puzzles," *Aging, Neuropsychology, and Cognition*, vol. 2, no. 2, pp. 146–155, 1995.
[91] J. L. Cohen, "The effect of letter frequency on anagram solution times," *Psychonomic Science*, vol. 11, no. 2, pp. 79–80, 1968.
[92] S. Harter, "Pleasure derived from challenge and the effects of receiving grades on children's difficulty level choices," *Child Development*, pp. 788–799, 1978.
[93] B. Choi, A. Ward, Y. Li, J. Arguello, and R. Capra, "The effects of task complexity on the use of different types of information in a search assistance tool," *ACM Transactions on Information Systems (TOIS)*, vol. 38, no. 1, pp. 1–28, 2019.
[94] M. McGregor, L. Azzopardi, and M. Halvey, "A systematic review of cost, effort, and load research in information search and retrieval, 1972–2020," *ACM Transactions on Information Systems*, vol. 42, no. 1, pp. 1–39, 2023.
[95] J. Platt *et al.*, "Probabilistic outputs for support vector machines and comparisons to regularized likelihood methods," *Advances in large margin classifiers*, vol. 10, no. 3, pp. 61–74, 1999.
[96] R. Raimondi, N. Tzoumas, T. Salisbury, S. Di Simplicio, and M. R. Romano, "Comparative analysis of large language models in the royal

college of ophthalmologists fellowship exams," *Eye*, vol. 37, no. 17, pp. 3530–3533, 2023.

[97] D. Johnson, R. Goodman, J. Patrinely, C. Stone, E. Zimmerman, R. Donald, S. Chang, S. Berkowitz, A. Finn, E. Jahangir, *et al.*, "Assessing the accuracy and reliability of ai-generated medical responses: an evaluation of the chat-gpt model," *Research square*, 2023.

[98] Y. Lu, M. Bartolo, A. Moore, S. Riedel, and P. Stenetorp, "Fantastically ordered prompts and where to find them: Overcoming few-shot prompt order sensitivity," in *Proceedings of the 60th Annual Meeting of the Association for Computational Linguistics (Volume 1: Long Papers)*, pp. 8086–8098, 2022.

[99] M. Sclar, Y. Choi, Y. Tsvetkov, and A. Suhr, "Quantifying language models' sensitivity to spurious features in prompt design or: How i learned to start worrying about prompt formatting," *The Twelfth International Conference on Learning Representations*, 2023.

[100] Z. X. Yong, R. Zhang, J. Forde, S. Wang, A. Subramonian, H. Lovenia, S. Cahyawijaya, G. Winata, L. Sutawika, J. C. B. Cruz, *et al.*, "Prompting multilingual large language models to generate code-mixed texts: The case of south east asian languages," in *Proceedings of the 6th Workshop on Computational Approaches to Linguistic Code-Switching*, pp. 43–63, 2023.

[101] H. Ma, C. Zhang, Y. Bian, L. Liu, Z. Zhang, P. Zhao, S. Zhang, H. Fu, Q. Hu, and B. Wu, "Fairness-guided few-shot prompting for large language models," *Advances in Neural Information Processing Systems*, vol. 36, 2024.

[102] N. Zhang, L. Li, X. Chen, S. Deng, Z. Bi, C. Tan, F. Huang, and H. Chen, "Differentiable prompt makes pre-trained language models better few-shot learners," *International Conference on Learning Representations*, 2022.

[103] Z. Xu, C. Wang, M. Qiu, F. Luo, R. Xu, S. Huang, and J. Huang, "Making pre-trained language models end-to-end few-shot learners with contrastive prompt tuning," in *Proceedings of the Sixteenth ACM International Conference on Web Search and Data Mining*, pp. 438–446, 2023.

[104] Z. Shi and A. Lipani, "Don't stop pretraining? make prompt-based fine-tuning powerful learner," *Advances in Neural Information Processing Systems*, vol. 36, pp. 5827–5849, 2023.

[105] K. Zhou, J. D. Hwang, X. Ren, and M. Sap, "Relying on the unreliable: The impact of language models' reluctance to express uncertainty," *arXiv preprint arXiv:2401.06730*, 2024.

[106] R. T. McCoy, S. Yao, D. Friedman, M. Hardy, and T. L. Griffiths, "Embers of autoregression: Understanding large language models through the problem they are trained to solve," *arXiv preprint arXiv:2309.13638*, 2023.

[107] T. Kojima, S. S. Gu, M. Reid, Y. Matsuo, and Y. Iwasawa, "Large language models are zero-shot reasoners," *Advances in neural information processing systems*, vol. 35, pp. 22199–22213, 2022.

[108] K. M. Collins, A. Q. Jiang, S. Frieder, L. Wong, M. Zilka, U. Bhatt, T. Lukasiewicz, Y. Wu, J. B. Tenenbaum, W. Hart, *et al.*, "Evaluating language models for mathematics through interactions," *Proceedings of the National Academy of Sciences*, vol. 121, no. 24, p. e2318124121, 2024.

[109] L. Zhou, P. A. Moreno-Casares, F. Martínez-Plumed, J. Burden, R. Burnell, L. Cheke, C. Ferri, A. Marcoci, B. Mehrbakhsh, Y. Moros-Daval, *et al.*, "Predictable artificial intelligence," *arXiv preprint arXiv:2310.06167*, 2023.

[110] A. Kukla, "An attributional theory of choice," in *Advances in experimental social psychology*, vol. 11, pp. 113–144, Elsevier, 1978.

[111] J. G. Nicholls, "Achievement motivation: Conceptions of ability, subjective experience, task choice, and performance.," *Psychological review*, vol. 91, no. 3, p. 328, 1984.

[112] C. S. Dweck and J. Bempechat, "Children's theories of intelligence: Consequences for learning," in *Learning and motivation in the classroom*, pp. 239–256, Routledge, 2017.

[113] L. Fischer and P. Wollstadt, "Precision and recall reject curves for classification," *arXiv preprint arXiv:2308.08381*, 2023.

[114] K. Hendrickx, L. Perini, D. Van der Plas, W. Meert, and J. Davis, "Machine learning with a reject option: A survey," *Machine Learning*, pp. 1–38, 2024.

[115] C. Deng, Y. Zhao, X. Tang, M. Gerstein, and A. Cohan, "Investigating data contamination in modern benchmarks for large language models," in *Proceedings of the 2024 Conference of the North American Chapter of the Association for Computational Linguistics: Human Language Technologies (Volume 1: Long Papers)* (K. Duh, H. Gomez, and S. Bethard, eds.), (Mexico City, Mexico), pp. 8698–8711, Association for Computational Linguistics, June 2024.

[116] A. Srivastava, A. Rastogi, A. Rao, A. A. M. Shoeb, A. Abid, A. Fisch, A. R. Brown, A. Santoro, A. Gupta, A. Garriga-Alonso, *et al.*, "Beyond the imitation game: Quantifying and extrapolating the capabilities of language models," *Journal of Machine Learning Research*, 2023.

List of Figures

1 **Supplementary Figure 1: Analysis of the performance according to the used prompt.** Performance by prompt (split by **correct**, avoidant and **incorrect** results) of (top) GPT, (middle) LLaMA and (bottom) BLOOM models on the five benchmarks in Table 2. For science the transparent yellow bars at the bottom represent the random guess probability (25%). 5
2 **Supplementary Figure 2: Confusion matrices summarising the scoring quality**. The rows are the expert scoring (considered as ground truth) while the columns are labels classified by our algorithmic scoring method. 7
3 **Supplementary Figure 3: Questions of study S1.** An example of the S1 study with an instance from anagram use in the Concerto questionnaire. The grey input window is used to collect the response from the participants. 10
4 **Supplementary Figure 4: Questions of study S2.** Example of the survey used to ask participants to assess the response generated by an AI model. 11
5 **Supplementary Figure 5: Analysis of of respondents of study S1.** (Left) Histogram of the number of respondents per item. (Right) Histogram of the quantity of items per respondent ID (there are four questions per item). We see that 9 respondents only completed one item while there was one participant that completed 38 items, with most respondents completing between 6 and 23 items. . . . 12
6 **Supplementary Figure 6: Spearman correlations between the responses and time taken for the four questions of the study S1.** All four questions correlate strongly in their responses, with all the estimated responses having very high correlations, and Q2 having a bit less. Only time of Q2 is negatively correlated with the responses, which is consistent (easier the higher these values are), so difficult questions should take more time. Except for the times of Q3 and Q4, times are not very correlated in general. Left: ungrouped. Right: correlations calculated after grouping results per item. . 13
7 **Supplementary Figure 7: Comparison of the crowdsourced human assessment versus expert scoring in study S2.** Confusion matrices showing the crowdsourced human assessments compared against expert scoring (considered as ground truth). Per benchmark. 15
8 **Supplementary Figure 8: Comparison of the crowdsourced human assessment versus expert scoring of study S2 in percentages.** Same as Supplementary Figure 7 (per benchmark) but in percentages (row-wise). 15
9 **Supplementary Figure 9: Comparison of the crowdsourced human assessment versus expert scoring of study S2 considering family and benchmark.** Confusion matrices showing the crowdsourced human assessments compared against expert scoring (considered as ground truth). Detail by family and benchmark. 16
10 **Supplementary Figure 10: Comparison of the crowdsourced human assessment versus expert scoring of study S2 considering family and benchmark in percentages.** Same as Supplementary Figure 9 (per family and benchmark) but in percentages (row-wise). 16
11 **Supplementary Figure 11: Transformations between the raw intrinsic difficulties and the calibrated difficulties.** Calibration functions mapping raw intrinsic difficulty to human calibrated difficulty for addition (N=3142), anagram (N=1570), locality (N=2340), transforms (N=730), GPQA (N=544) and OpenBookQA (N=1000). The results of 30 and 5 equal-sized bins are also shown. For better visualisation, the x-axis of the subplot for locality has been applied a logarithmic (base 10) transformation and subtracted the minimum difficulty (-11.97), after the logistic fitting. 19
12 **Supplementary Figure 12: Prompting stability for the GPT family over difficulty.** Proportion of *correctness*, *avoidance* and *incorrectness* over difficulty for the 15 prompt templates for the GPT family. Layout and details as in Extended Data Fig. 3. 20
13 **Supplementary Figure 13: Prompting stability for the LLaMA family over difficulty.** Proportion of *correctness*, *avoidance* and *incorrectness* over difficulty for the 15 prompt templates for the LLaMA family. Layout and details as in Extended Data Fig. 3. 21
14 **Supplementary Figure 14: Performance of BLOOM models over difficulty.** Layout and details as in Figure 2 but for the BLOOM family. 22

15 **Supplementary Figure 15: Prompting stability for the BLOOM family over difficulty**. Proportion of *correctness*, *avoidance* and *incorrectness* over difficulty for the 15 prompt templates for the BLOOM family. Layout and details as in Extended Data Fig. 3. 23

16 **Supplementary Figure 16: Types of avoidance for each model-domain pair.** The proportions (%) of different kinds of avoidance across the models and domains, after annotating 4000 randomly extracted examples that are balanced across domains (i.e., 800 examples per domain) and models (i.e., an equal number of examples per model, whenever applicable). Empty cells represent model-domain pairs where zero avoidance was observed. Note that we have defined six avoidance types (one was not found in the sample, 'ethical avoidance (passive)'). 30
